# Minute-scale persistence of a GPCR conformation state triggered by non-cognate G protein interactions primes signaling

**DOI:** 10.1038/s41467-019-12755-9

**Published:** 2019-10-23

**Authors:** Tejas M. Gupte, Michael Ritt, Matthew Dysthe, Rabia U. Malik, Sivaraj Sivaramakrishnan

**Affiliations:** Department of Genetics, Cell Biology, and Development, University of Minnesota, Twin-Cities, Minneapolis, Minnesota 55455 USA

**Keywords:** Biochemistry, Hormone receptors, Pharmacology

## Abstract

Despite the crowded nature of the cellular milieu, ligand–GPCR–G protein interactions are traditionally viewed as spatially and temporally isolated events. In contrast, recent reports suggest the spatial and temporal coupling of receptor–effector interactions, with the potential to diversify downstream responses. In this study, we combine protein engineering of GPCR–G protein interactions with affinity sequestration and photo-manipulation of the crucial Gα C terminus, to demonstrate the temporal coupling of cognate and non-cognate G protein interactions through priming of the GPCR conformation. We find that interactions of the Gαs and Gαq C termini with the β_2_-adrenergic receptor (β_2_-AR), targeted at the G-protein-binding site, enhance Gs activation and cyclic AMP levels. β_2_-AR–Gα C termini interactions alter receptor conformation, which persists for ~90 s following Gα C terminus dissociation. Non-cognate G-protein expression levels impact cognate signaling in cells. Our study demonstrates temporal allostery in GPCRs, with implications for the modulation of downstream responses through the canonical G-protein-binding interface.

## Abstract (web summary)

Recent reports suggest the spatial and temporal coupling of receptor-effector interactions, with the potential to diversify downstream responses. Here authors demonstrate the temporal coupling of cognate and non-cognate G protein interactions through priming of GPCR conformation.

## Introduction

GPCR signaling occurs in a crowded cellular environment that juxtaposes multiple GPCRs and G proteins/effector molecules^1^. Nonetheless, the traditional model of GPCR signaling views the efficacy of effector activation by a GPCR as an isolated event that is spatially and temporally separated from the cellular milieu^2–4^. In contrast, emerging data suggest that GPCR–effector interactions can be spatially and temporally coupled, thereby enhancing the context-dependent multiplicity inherent in physiological signaling pathways. For instance, the prototypical GPCR opsin was shown to retain an active conformation on the time scale of minutes following ligand dissociation^5,6^, potentially coupling the activation of multiple G proteins to a single ligand-binding event. The β_2_ adrenergic receptor (β_2_-AR) can bind to both β-arrestin and Gs simultaneously, leading to enhanced cAMP activation that contrasts with the canonical view of sequential binding of these effectors^7^. Lateral interaction between class A GPCRs can lead to oligomerization that can reinforce signaling through the cognate pathway^8^. Hence, probing the molecular basis of spatial and temporal coupling of GPCR–effector interactions will significantly advance our understanding of signaling in a cellular context.

We recently reported that G protein interactions with a GPCR synergistically increase downstream signaling, a phenomenon we termed GPCR priming^9^. Here we dissect the spatial and temporal connections between events that lead to GPCR priming. Given that the outcome of GPCR–G protein interactions is dependent on expression levels^10,11^ and spatial localization^12,13^, we used the established Systematic Protein Affinity Strength Modulation (SPASM) technique to control and modulate the effective concentration within these interactions in live cells^14^. SPASM employs an ER/K α-helical linker to tether and modulate the effective concentration between two polypeptides, without enforcing interactions^15^. We have repeatedly demonstrated the utility of this technique in monitoring and modulating GPCR–G protein interactions under basal conditions and in response to agonist stimuli^9,16^. A variation of this technique controls and probes the interaction between the GPCR and the C terminus of the Gα subunit^14,17^, a known determinant of GPCR–G protein coupling efficacy and specificity^18,19^. In addition to SPASM sensors, we employ affinity sequestration and UV irradiation of synthetic, photocleavable (PC) Gα C-terminal peptides to investigate the temporal regulation of GPCR–G protein interactions. Taken together, these approaches delineate the sequence of events underlying GPCR priming.

In this study, we use a prototypical Gs-coupled receptor, β_2_-AR, to investigate the sequence of interactions during GPCR priming. The presence of either the cognate (Gs) or non-cognate (Gq) Gα C-terminal peptides enhances the β_2_-AR–Gαs C terminus interaction, Gs activation, and cellular cyclic AMP levels. Swapping hotspot residues that dictate Gα C terminus interactions at the canonical G-protein-binding site on the receptor switch the profile of cognate and non-cognate responses. Cognate and non-cognate Gα C-terminal peptides alter β_2_-AR conformation, which persists for ~90 s following affinity sequestration (biotinylated peptide) or photocleavage (PC peptide). β_2_-AR demonstrates enhanced Gs activation, despite removal of the non-cognate Gα C-terminal peptide. Together, these observations suggest that non-cognate G proteins can prime receptor conformation through weak interactions at the canonical G-protein-binding site. Following dissociation of the non-cognate G protein, the primed receptor conformation persists for ~90 s leading to enhanced cognate G-protein activation and downstream signaling. This model suggests that non-cognate G-protein expression levels can influence cognate responses. Accordingly, overexpression of Gαq in HEK293 cells augments fenoterol-induced cyclic AMP levels. Our study highlights the interconnected nature of GPCR–G protein interactions and identifies the potential for temporal allosteric modulation of GPCR signaling through the canonical G-protein-binding interface.

## Results

### Hotspot residues in Gα peptides dictate magnitude of priming

**Fig. 1:**
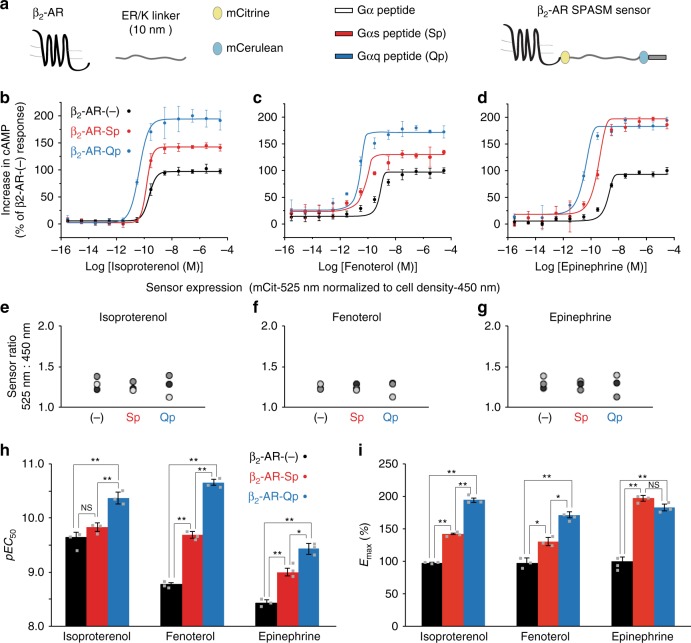
Gα peptides potentiate the cAMP response to different β_2_-AR agonists. **a** Schematic list of proteins and protein domains used to assemble SPASM sensors linking β_2_-AR to cognate (Sp) and non-cognate (Qp) Gα C terminus peptides. cAMP accumulation in HEK293T cells expressing equivalent amounts of β2-AR SPASM sensors. Cells expressing either control (−) or peptide sensors (Sp/Qp) were stimulated with varying concentrations (0.3 fM to 30 μM) of isoproterenol (**b**), fenoterol (**c**), or epinephrine (**d**), and the cAMP responses measured. The increase in cAMP is expressed as a percentage, normalized to the cAMP response observed from β2-AR-(-) expressing cells at saturating concentration of the indicated agonist. **e**–**g** Normalized mCit fluorescence intensities (em 525  nm/em 450 nm) confirm equivalent sensor expression, for each agonist condition. **h** The cAMP response data were fit to a four-parameter logistic function to obtain concentration–response curves and estimate potency (*pEC*_50_). **i** For each agonist, the maximum cAMP response (*E*_max_) at saturating agonist concentration was compared across the three sensors. Values are mean ± SD from at least nine repeats across three experiments (**b**–**d**). The means from each experiment are indicated (gray squares). Statistically significant differences were assessed by a one-way ANOVA, followed by Tukey’s post-hoc test. Significance is denoted by asterisks, NS-not significant, *p *<* 0.05; **p < 0.01

The critical role of the Gα-subunit C terminus peptide (Gα peptide) in GPCR–G protein interactions is well-established^18–20^. We have recently demonstrated that native peptides from the Gα C terminus are sufficient for GPCR priming^9^. Specifically, peptides comprising the entire α5 helix (27 amino acids)^21^ of the non-cognate Gα subunit (Gαq–Qp) stimulate greater second messenger levels upon isoproterenol stimulation compared with their cognate counterparts (Gαs–Sp), although untransfected cells exhibit meager cAMP response relative to the β_2_-AR-(-) sensor-overexpressing cells^9^. Here we use concentration–response curves to compare the cAMP response with different β_2_-AR agonists (Fig. 1a-d) from cells with equivalent SPASM sensor expression (Fig. 1e-g). We find that tethering of Qp or Sp to β_2_-AR in live cells using a SPASM sensor enhanced potency (EC_50_) and *E*_max_ with isoproterenol, the β_2_-AR-selective ligand fenoterol^22^, and the endogenous agonist epinephrine (Fig. 1h, i), recapitulating GPCR priming. All three ligands show a significant increase in pEC_50_ (Fig. 1h) in the presence of non-cognate Qp, whereas Sp increases the potency of fenoterol and epinephrine. We find ligand-specific increases in *E*_max_ for Qp and Sp for all the three ligands (Fig. 1i), such that only isoproterenol and fenoterol show significant differences between the non-cognate and cognate Gα peptides. Hence, we used isoproterenol and fenoterol to gain insight into the GPCR–Gα peptide-binding site responsible for enhanced signaling.

**Fig. 2:**
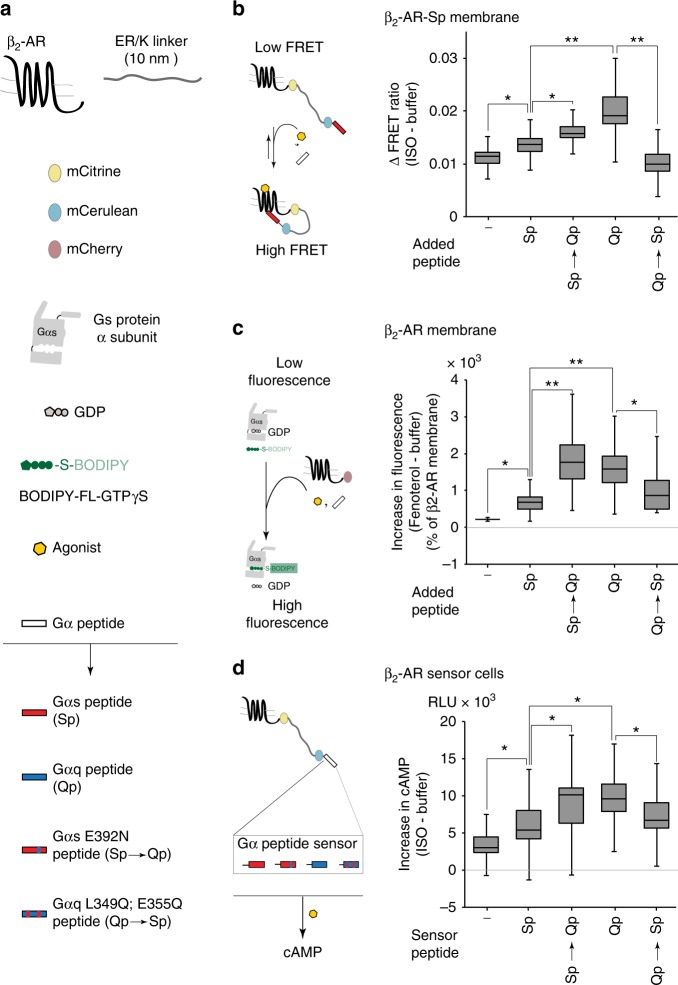
Switching receptor-interacting hotspot residues influences β_2_-AR priming. **a** Schematic list of proteins and protein domains used to assemble sensors for β_2_-AR and G-protein activation. The Gα peptides used are Sp (cognate), Qp (non-cognate), Sp → Qp (Sp mutant E392N, mimicking Qp), and Qp → Sp (Qp mutant L349Q, E355Q, mimicking Sp). **b** Left, schematic representation of an assay for using FRET to detect agonist-stimulated β_2_-AR activation using native membranes from HEK293 cells expressing β_2_-AR-Sp SPASM sensor in the presence of Gα peptides. Right, change in FRET ratio (mCit/mCer) following isoproterenol treatment (100 μM) of β_2_-AR-Sp sensors in the presence of the indicated Gα peptides. Qp and Sp → Qp peptide result in increased β_2_-AR activation. **c** Left, assay schematic for measuring G-protein activation by agonist-stimulated β_2_-AR in the presence of Gα peptides using BODIPY-GTPγS. Right, G-protein activation measured from the increase in BODIPY-FL-GTPγS fluorescence following fenoterol treatment (10 μM, 3 min) in urea-stripped membranes from HEK293T cells expressing β_2_-AR-mCherry, and containing purified Gαs (100 nM) and the indicated Gα peptides (10 μM). Values are expressed relative to G-protein activation by membrane in the absence of peptide (−). Qp and Sp → Qp peptide result in enhanced Gαs activation. **d** Left, SPASM sensors, tethering β_2_-AR to the individual Gα peptides, used for transfection into HEK293T cells. Cells expressing equivalent amount of sensor were analyzed for agonist-stimulated downstream signalling. Right, cAMP accumulation in cells expressing equivalent amounts of the indicated β_2_-AR–Gα peptide SPASM sensors following stimulation with isoproterenol (ISO, 10 μM) for 5 min. Qp and Sp → Qp peptide sensors enhance cAMP levels. Box-and-whisker plots: center line is median, box ends are upper and lower quartiles, whisker ends are 1.5 × interquartile range (IQR) from four independent experiments with at least three replicates per experiment (*n* ≥ 4). Statistically significant differences were assessed by a one-way ANOVA, followed by Tukey’s post-hoc test. Significance is denoted by asterisks, *p < 0.05; **p < 0.01

We have previously identified three hotspot residues in the Gα peptide that influence downstream signaling through their interactions with the GPCR at the well-characterized, canonical G-protein-binding site^17^. This site on the GPCR is the primary interface for interaction with G proteins^18,19,23^. Hence, we used site-directed mutagenesis to swap hotspot residues in Sp (Sp → Qp) and Qp (Qp → Sp) that should switch the specificity of receptor interaction at the canonical G-protein-binding site. We examined the effect of hotspot residue substitution at the level of receptor conformation, G-protein activation, and downstream signaling, using three different assays. First, we probed receptor conformation using a SPASM sensor that measures the strength of interaction between β_2_-AR and its cognate Gαs peptide (Sp)^14^. Changes in fluorescence resonance energy transfer (FRET) ratio (ΔFRET) of SPASM sensors correlate linearly with the strength of interaction of proteins/polypeptides flanking the ER/K linker^24^. Membrane preparations containing the SPASM sensor, pre-incubated with Qp, showed significantly greater ΔFRET compared with Sp (Fig. 2b). Swapping hotspot residues decreased non-cognate (Qp → Sp < Qp) and increased cognate (Sp → Qp > Sp) responses. Second, we examined Gs activation using a previously described in vitro assay that combines urea-stripped membranes overexpressing mCherry-tagged β_2_-AR, recombinant Gs protein, and Gα peptides^9^. Fenoterol stimulation triggered greater Gs activation for Qp compared with Sp (Fig. 2c), consistent with our previous observations^9^. Swapping hotspot residues decreased non-cognate (Qp → Sp < Qp) and increased cognate (Sp → Qp > Sp) Gs activation levels. Third, we measured cAMP generation in live HEK293 cells expressing β_2_-AR SPASM sensors, which provide pairwise control on the receptor–Gα peptide interaction^14^ (Fig. 2d). Tethering Qp increased cAMP over Sp, whereas hotspot residue substitutions switched the response profile (Qp → Sp < Qp and Sp → Qp > Sp). The influence of cognate and non-cognate Gα peptides on G-protein activation and cAMP response from β_2_-AR also translated to the V_1a_ vasopressin receptor (Supplementary Fig. 1a, b). Swapping hotspot residues decreased non-cognate (Sp → Qp < Sp) and increased cognate (Qp → Sp > Qp) G-protein activation and IP_1_ levels. For signaling assays downstream of β_2_-AR and V_1a_-R, sensors were expressed at equivalent levels as assessed by the mCitrine fluorescence (Supplementary Fig. 1c, d). Taken together, these data support a possible role for GPCR–Gα peptide interactions at the canonical G-protein-binding site in GPCR priming.

### Strength and stability of the β_2_-AR–Gα peptide interaction

**Fig. 3:**
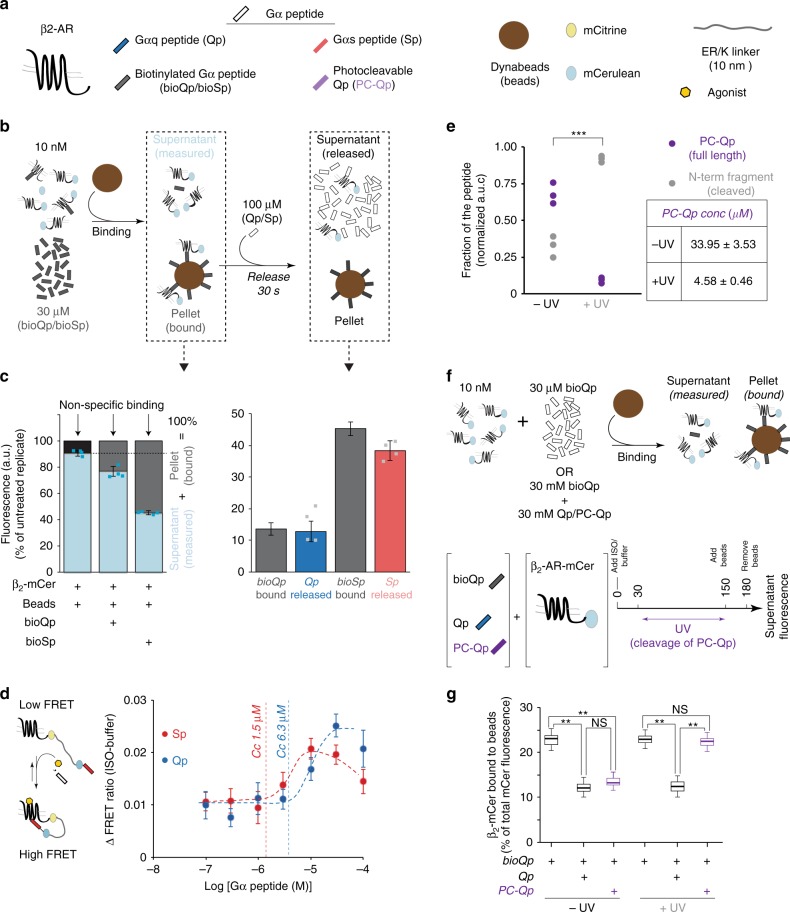
Weak, transient interactions with Gα peptides increase β_2_-AR priming. **a** Schematic list of reagents used to measure Gα peptide–GPCR interaction. **b** Representation of affinity pull-down and release assay for monitoring the interaction strength of Qp with β_2_-AR-mCer. **c** mCer fluorescence quantification of supernatants estimate extent of bioQp and bioSp binding to the receptor. Only 13% of β_2_-AR-mCer was specifically pelleted by binding to bioQp and all the bound receptors were completely released by excess Qp in 30 s. bioSp bound to 45% of the receptor and most of it was released by an excess of the Sp. Values are mean ± SD from three independent experiments with three repeats per experiment (*n* = 3). **d** Left, ΔFRET assay for agonist-stimulated β_2_-AR activation, in the presence of Gα peptides, using native β_2_-AR-Sp sensor membranes. Right, measuring the effect of Gα peptide (Sp/Qp) concentration on ISO-stimulated (100 μM) ΔFRET ratio of β_2_-AR-Sp sensors. Critical concentration (*C*_C_) to elicit response is indicated. Values are mean ± SD from four independent experiments with at least four repeats per experiment (*n* ≥ 4). **e** Quantification of the extent of photocleavage of PC-Qp by MALDI-MS. UV irradiation of full-length PC-Qp in the presence of native membranes causes the concentration of the full-length PC-Qp to decrease to 4.58 μM, which is less than the critical concentration (*C*_C_) required for GPCR priming. Values are mean ± SD from three experiments. **f** Representation of a competition assay to monitor displacement of PC-Qp (fragments) from native membranes expressing β_2_-AR-mCer. **g** Presence of Qp or intact PC-Qp competes with bioQp for binding to the receptor. Thirty seconds after UV irradiation, PC-Qp no longer competes with bioQp for interaction with the receptor. Values are from at least 12 replicates across 3 experiments Box-and-whisker plots: center line is median, box ends are upper and lower quartiles, whisker ends are 1.5 × interquartile range (IQR) from four independent experiments with at least three replicates per experiment (*n* ≥ 4). Statistically significant differences were assessed by a one-way ANOVA, followed by Tukey’s post-hoc test. Significance is denoted by asterisks, NS-not significant, ***p < 0.001, **p < 0.01

We have previously reported that we can detect an isoproterenol-stimulated interaction between the cognate Sp and β_2_-AR, but not between the non-cognate Qp and β_2_-AR in live cells using the respective GPCR SPASM sensors^14^. However, Sp primes β_2_-AR to a lesser extent than Qp (Fig. 1). To understand the differences in the priming ability of Sp and Qp, we developed an affinity sequestration assay to compare the strength and stability of the β_2_-AR interaction with both the Gα peptides (Fig. 3a-c). Streptavidin-coated magnetic beads were used to pull down the Gα peptide using an N-terminal biotin tag (bioSp/bioQp) and were found to remove over 95% of bioQp (30 μM → 1.6 μM) (bioQp, Supplementary Fig. 2a). The strong interaction between magnetic beads and biotinylated Gα peptide was used to determine the bimolecular dissociation constant (*K*_D_) of the interaction between Gα peptide and β_2_-AR, by a selective pull down of β_2_-mCer in complex with the peptide (Fig. 3b, c). An excess of either one of the biotinylated Gα peptides (30 μM) was incubated with a known concentration of β_2_-AR (10 nM β_2_-mCer expressing membranes) and the peptide–receptor complex was depleted through magnetic beads (Fig. 3b). The unbound receptor was evaluated from the supernatant fluorescence. In the absence of biotinylated Gα peptide, the magnetic beads exhibited some nonspecific interaction with the β_2_-mCer-expressing membranes, accounting for ~10% of receptor (Supplementary Fig. 2b and Fig. 3c). Interaction with bioQp resulted in the specific pull down of 13% receptor, suggesting a weak interaction (*K*_*D*_ *>* 200 μM; Fig. 3b, c). Under similar conditions, interaction with bioSp resulted in 45% of β_2_-mCer being pulled down specifically by the magnetic beads, indicating a *K*_D_ ~30 μM (Fig. 3b, c). The β_2_-mCer pelleted by the biotinylated Gα peptide was competitively displaced from the magnetic beads within 30 s following the addition of an excess of the corresponding unlabeled peptide (100 μM) (Fig. 3b, c), indicating the transient nature of these interactions.

The synergies in cognate and non-cognate Gα-peptide interactions were further evaluated from the changes in the interaction between β_2_-AR and the tethered Sp, in the presence of increasing concentrations of unlabeled Qp and Sp in solution (Fig. 3d). Sp and Qp enhanced the FRET response in a concentration-dependent manner. Consistent with the stronger β_2_-AR-Sp interaction compared with Qp, lower concentrations of unlabeled Sp compared with Qp are necessary to augment the sensor response (critical concentration (*C*_C_) for enhanced FRET = 1.5 μM for Sp; 6.3 μM for Qp). However, at higher concentrations ( > 15 μM), Sp, but not Qp, shows a diminished response consistent with the competition between the sensor and unlabeled peptides. Comparison of the FRET response curves for Sp and Qp also reveals that Sp has lower maximum amplitude than Qp. Thus, the β_2_-AR-Qp interaction is much weaker relative to the β_2_-AR-Sp interaction, suggesting that weaker interactions have a greater ability to prime the receptor.

The weak and transient nature of the interaction between Qp and β_2_-AR was further confirmed using PC-Qp (Fig. 3e-g). Tyrosine at position 356 in Qp was replaced by the photolabile derivative, Anp [3-amino-3-(2-nitrophenyl)-propionic acid], such that the resulting PC-Qp was cleaved into N-term and C-term fragments upon irradiation with UV (Supplementary Fig. 3a). Irradiation for 2 min reduced the amount of full-length PC-Qp to 4.58 μM as determined by mass spectrometry (MS) (Fig. 3e and Supplementary Fig. 3b), which is less than the critical concentration of Qp required for priming (6.3 μM). The ability of the PC-Qp fragments to bind to the receptor was monitored by a competition assay (Fig. 3f, g). As observed for the bioQp association experiments (Fig. 3b, c), an excess of bioQp (30 μM) led to the pulldown of a fraction of β_2_-mCer receptor (~20%) by streptavidin-coated magnetic beads. Addition of either Qp (30 μM) or PC-Qp (50 μM) competed with bioQp for complexing the receptor (Fig. 3g, −UV). However, irradiation of PC-Qp before adding the magnetic beads abrogated the ability of PC-Qp to compete with bioQp (Fig. 3g, +UV). This indicates that the fragments generated by cleavage of PC-Qp do not compete with bioQp for binding to the receptor. Thus, the fragments of PC-Qp have dissociated from the receptor within the time course of the irradiation regime (2 min) (Fig. 3f). Therefore, the UV-irradiated PC-Qp is unlikely to persistently interact with β_2_-AR. In addition, PC-Qp-stimulated FRET levels were similar to unlabeled Qp, albeit a higher concentration (50 μM PC-Qp vs. 30 μM Qp) was necessary likely due to the cleavage of PC-Qp despite experimental precautions to prevent UV irradiation (Supplementary Fig. 3b, c).

The ability of the Gα peptides to exert GPCR priming, despite weak and transient interactions indicates that the effect of GPCR priming may be observed even after the dissociation of the GPCR–Gα peptide interaction, suggesting a temporal dimension to the priming phenomenon. As Qp has a greater effect on priming β_2_-AR, we focused on this interaction.

### Temporal persistence of β_2_-AR activation state

**Fig. 4:**
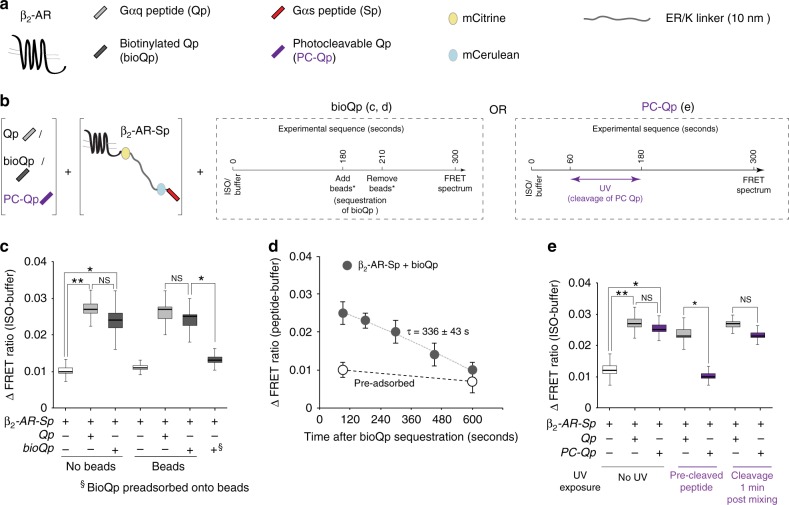
β2-AR conformation persists following interactions with non-cognate Qp. **a** Schematic list of reagents used to measure Gα peptide–GPCR interaction. **b** Schematic of ΔFRET assay to monitor time dependence of agonist-stimulated β_2_-AR activation, after depletion of Qp; center, sequestration of bioQp using Dynabeads; right, cleavage of PC-Qp using UV exposure. **c** ISO (100 μM)-induced ΔFRET ratio of β_2_-AR-Sp sensors (in native membranes) in the presence or absence of Gα peptides, with and without treatment involving Dynabeads that sequester bioQp. § indicates treatment of membranes with supernatant from bioQp preadsorbed onto Dynabeads. **d** ΔFRET ratio of β_2_-AR-Sp sensors at increasing time after sequestration of bioQp. ISO (100 μM)-treated β_2_-AR-Sp sensors were exposed to bioQp (30 μM) or buffer control for 3 min, followed by Dynabeads for 30 s. FRET spectra were monitored at indicated times after bioQp sequestration by Dynabeads. Open symbols indicate treatment with supernatant of bioQp preadsorbed by Dynabeads. Values are mean ± SD from three independent experiments with at least four replicates per experiment (*n* ≥ 3). **e** ISO (100 μM)-induced ΔFRET ratio of β_2_-AR-Sp sensors in the presence or absence of Gα peptides (Qp- 30 μM or PC-Qp- 50 μM), with and without UV treatment that cleaves PC-Qp, but does not affect Qp. Photocleavage of PC-Qp before addition to the membrane (pre-cleaved) abrogates the ΔFRET response relative to Qp (not photolabile), whereas photocleavage after mixing PC-Qp with the membrane maintains the enhanced ΔFRET response. Box-and-whisker plots: center line is median, box ends are upper and lower quartiles, whisker ends are 1.5 × interquartile range (IQR) from four independent experiments with at least three repeats in replicates per experiment (*n* ≥ 4). Statistically significant differences were assessed by a one-way ANOVA, followed by Tukey’s post-hoc test. Significance is denoted by asterisks, NS-not significant, *p < 0.05; **p < 0.01

We have previously found that the β_2_-AR-Sp SPASM sensor reports on the activation state of the GPCR^14^. Hence, the readout from this sensor was used to probe for temporal persistence of β_2_-AR activation following removal of non-cognate Gα peptide (Qp). We used two orthogonal strategies to remove Qp: affinity sequestration (Fig. 4a-d) and photocleavage (Fig. 4a, b, e). β_2_-AR-Sp sensor-containing membranes were treated with either a biotinylated (bio) or a PC version of Qp, which resulted in an increased FRET response when stimulated with isoproterenol (Fig. 4 c, e). Pre-depletion of bioQp or pre-cleavage of PC-Qp to levels below the critical concentration (*C*_C_; Supplementary Fig. 2 and Fig. 3d) prior to mixing with membranes resulted in FRET responses indistinguishable from membranes without peptide addition. However, depletion (bioQp) or photocleavage (PC-Qp) following treatment with isoproterenol (timeline, Fig. 4b) resulted in a persistent FRET response, with an increase in magnitude relative to the absence of peptide. This FRET response was sustained for 90 s (bioQp, Fig. 4c) and 120 s (PC-Qp, Fig. 4e) after peptide removal. Monitoring the FRET response of bioQp-depleted membranes over time shows a linear decay to control membranes (not treated with peptides), with a half-time (*τ*) of 336 s (Fig. 4d). Together, these data show that although the interactions between non-cognate Gα peptide and GPCR are weak and transient, they can cause a persistent increase in the agonist-activated state of the receptor.

### Temporal persistence of changes in β_2_-AR conformation

**Fig. 5:**
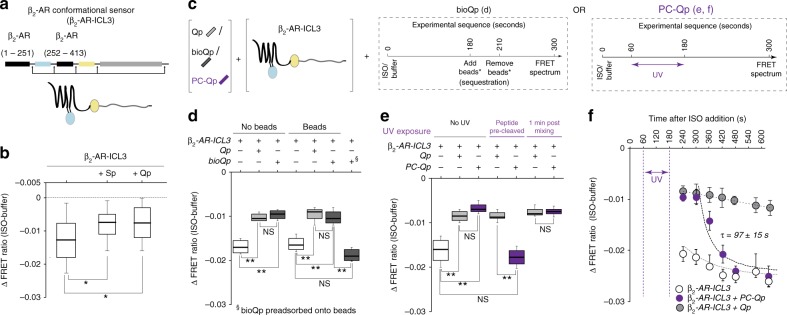
Temporal persistence of β_2_-AR conformational changes following Qp sequestration. **a** Design of β_2_-AR conformational sensor (β_2_-AR-ICL3), based on intramolecular FRET between mCer (donor) located in ICL3 and mCit (acceptor) inserted at the C terminus of the receptor. **b** Agonist-stimulated changes in β_2_-AR conformation monitored by ΔFRET ratio using native membranes containing β_2_-AR-ICL3 sensor, in the presence or absence of Gα peptides. **c** Schematic of ΔFRET assay to monitor time dependence of agonist-stimulated changes in β_2_-AR conformation, using native β_2_-AR-ICL3 sensor membranes, after depletion of Qp; center, sequestration of bioQp using Dynabeads; right, cleavage of PC-Qp using UV exposure. **d** ISO (100 μM)-induced ΔFRET ratio of β_2_-AR-ICL3 sensors in the presence or absence of Gα peptides, with and without treatment with Dynabeads that sequester bioQp. § indicates treatment of membranes with supernatant from bioQp preadsorbed onto Dynabeads. **e** ISO (100 μM)-induced ΔFRET ratio of β_2_-AR-ICL3 sensors in the presence or absence of Gα peptides (Qp- 30 μM or PC-Qp- 50 μM), with and without UV treatment that cleaves PC-Qp, but does not affect Qp. Photocleavage of PC-Qp before addition to membrane (pre-cleaved) abrogates the ΔFRET response relative to Qp (not photolabile), whereas photocleavage after mixing PC-Qp with membrane maintains the ΔFRET response. **f** Temporality of agonist-induced ΔFRET ratio of β_2_-AR-ICL3. Membranes containing the β_2_-AR-ICL3 sensor mixed with either no peptide (open circles), Qp (30 μM; grey circles), or PC-Qp (50 μM; purple circles) were treated with either ISO (100 μM) or buffer for 1 min, followed by UV exposure for 2 min. FRET spectra were acquired at increasing time intervals, starting at 30 s after UV exposure (210 s) and ΔFRET ratio calculated. Values are mean ± SD from three independent experiments with at least four replicates per experiment (*n* ≥ 3). Box-and-whisker plots: center line is median, box ends are upper and lower quartiles, whisker ends are 1.5 × interquartile range (IQR) from four independent experiments with at least three repeats in replicates per experiment (*n* ≥ 4). Statistically significant differences were assessed by a one-way ANOVA, followed by Tukey’s post-hoc test. Significance is denoted by asterisks, NS-not significant, *p < 0.05; **p < 0.01

To investigate the structural basis for the persistence of β_2_-AR priming, we next used a FRET-based conformational sensor designated β_2_-AR-ICL3 (Fig. 5a), engineered on the basis of previous designs^25,26^. β_2_-AR-ICL3 encodes a functional receptor, displaying agonist-stimulated cAMP response (Supplementary Fig. 4). β_2_-AR-ICL3 reports agonist binding as a decrease in FRET ratio of sensor-containing membranes (Fig. 5b). Addition of either unlabeled Sp or Qp to β_2_-AR-ICL3 sensor membranes reduced the magnitude of the FRET response to isoproterenol. Thus, stimulation by a combination of either of the Gα peptides along with an agonist induces distinct conformational changes in β_2_-AR, compared with agonist-mediated activation. We next determined whether the changes in β_2_-AR conformation, induced by the combination of Qp and isoproterenol, also persist over time. As in the case of receptor activation, temporal persistence of receptor conformation was monitored from the changes in the FRET response, following removal of Qp by affinity sequestration (Fig. 5c, d) or photocleavage (Fig. 5c, e, f). β_2_-AR-ICL3 sensor-containing membranes were treated with either a biotinylated (bio) or a PC version of Qp, which attenuated the FRET response to isoproterenol (Fig. 5d, e). BioQp and PC-Qp stimulated FRET levels were similar to unlabeled Qp. Pre-depletion of bioQP or pre-cleavage of PC-Qp to levels below the critical concentration (*C*_C_; Supplementary Fig. 2 and Fig. 3d) prior to mixing with the membranes resulted in FRET responses similar to membranes without peptide addition. However, following treatment with isoproterenol (timeline Fig. 5c), depletion (bioQp) or photocleavage (PC-Qp) resulted in a persistent FRET with a reduced magnitude, relative to the absence of peptide. This FRET response was sustained for 90 s (bioQp, Fig. 5d) and 120 s (PC-Qp, Fig. 5e) after peptide removal. Time course of FRET response of PC-Qp-depleted membranes shows an exponential decay to levels for membranes not treated with peptides, with a half-time (*τ*) of 97 s (Fig. 5f). Together, these data support a Gα peptide-stimulated change in β_2_-AR conformation that persists for at least 2 min after peptide removal.

### Temporal persistence of G-protein activation

**Fig. 6:**
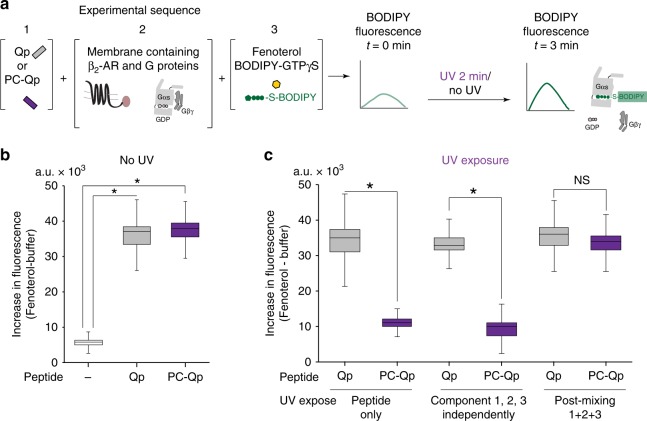
Persistence of G-protein activation after PC-Qp cleavage. **a** Assay schematic for measuring G-protein activation by agonist-stimulated β_2_-AR in the presence of Gα peptides. G-protein activation levels measured from the increase in BODIPY-FL-GTPγS fluorescence following fenoterol treatment (10 μM, 3 min) of native membranes from HEK293T cells expressing β_2_-AR-mCherry, with endogenous Gs and the indicated Gα peptides (10 μM). **b** In the absence of UV treatment, Qp (30 μM) or PC-Qp (50 μM) cause an equivalent increase in BODIPY-FL-GTPγS fluorescence. **c** Photocleavage of PC-Qp before addition to the membrane (peptide only, or components 1, 2, 3 independently) suppresses G-protein activation. UV treatment of Qp (not photolabile) displays enhanced G-protein activation similar to untreated Qp. Photocleavage of PC-Qp post-mixing does not abrogate G-protein activation. Box-and-whisker plots: center line is median, box ends are upper and lower quartiles, whisker ends are 1.5 × IQR (interquartile range) from three independent experiments with three technical replicates per experiment (*n* **=** 3). Statistically significant differences were assessed by one-way ANOVA followed by post-hoc Tukey’s test. NS indicates not significant and significance of *p* < 0.05 is indicated by *

We next examined whether the temporal persistence of β_2_-AR conformation and activation state translate to G-protein activation. Cell membranes expressing β_2_-AR-mCherry were stimulated with fenoterol and the activation of endogenous membrane-associated Gs protein was monitored using BODIPY-FL-GTPγS in the absence or presence of exogenously added, unlabeled non-cognate peptides (Fig. 6a). G-protein activation in response to fenoterol was dramatically increased upon addition of either Qp or PC-Qp (~7-fold increase) (Fig. 6b), similar to Qp-stimulated G-protein activation observed earlier (Fig. 2c). This primed response was lost when PC-Qp was exposed to UV before mixing with other reactants. Interestingly, when membranes were exposed to UV after the addition of PC-Qp, enhanced G-protein activation persisted for 120 s after photocleavage of the peptide (Fig. 6c). Having uncovered that temporal persistence is an underlying theme of priming β_2_-AR, we sought to examine its influence on cellular signaling.

### Physiological implications of β_2_-AR priming

**Fig. 7:**
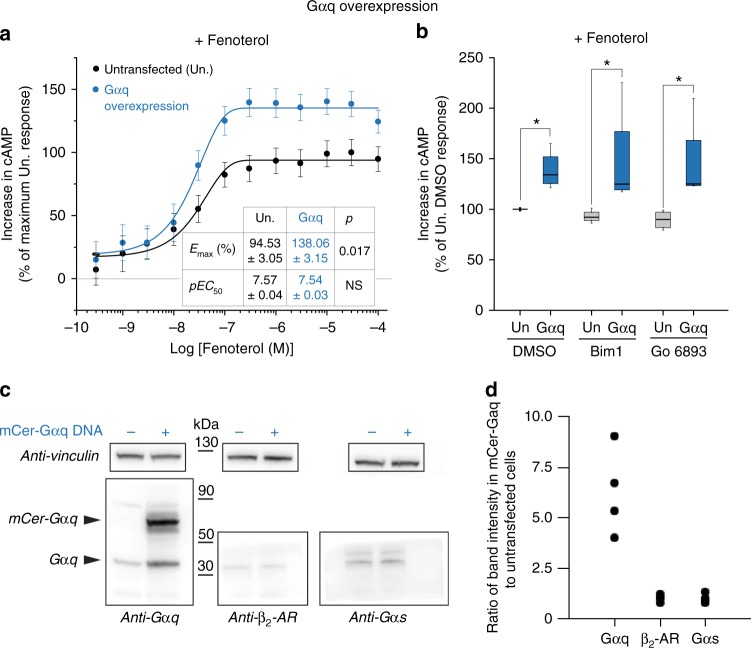
Gαq overexpression enhances cAMP response of endogenous β_2_-AR. **a** cAMP accumulation in HEK293T cells either overexpressing Gαq (mCer-tag) or an equivalent number of untransfected control cells following stimulation with increasing concentration of fenoterol (0.3 nM to 100 μM) in the presence of 2 μM forskolin (see Methods). The increase in cAMP with fenoterol concentration was fit to a four-parameter logistic function to estimate EC_50_ and *E*_max_ (Methods). Values are mean ± SD from six experiments with at least three repeats per experiment (*n* ≥ 6). Statistically significant differences in the EC_50_ and *E*_max_ values were assessed by Students’ *t*-test. **b** cAMP accumulation in HEK293T cells either overexpressing Gαq (mCer-tag) or equivalent number of untransfected control cells, following stimulation with fenoterol (10 μM) and forskolin (2 μM), in the presence of PKC inhibitors Bim1 (1 μM) or Gö6983 (1 μM), or DMSO. Cells overexpressing Gαq had accumulated greater cAMP even in the presence of PKC inhibitors. The drug treatments do not influence the cAMP response across the three untransfected or the three transfected conditions. Box-and-whisker plots: center line is median, box ends are upper and lower quartiles, whisker ends are 1.5 × IQR (interquartile range) from four independent experiments with three technical replicates per experiment (*n* ≥ 4). Statistically significant differences were assessed by two-way ANOVA followed by Tukey’s post-hoc test. Significance of *p* *<* 0.05 is indicated by *. **c** Western blot analysis of lysates from equivalent cell numbers of Gαq overexpression ( + ) and untransfected cells ( − ). The blottings were probed with the indicated antibodies with anti-vinculin serving as loading control. **d** Effect of Gαq overexpression on the levels of indicated proteins is expressed as the ratio of band intensity in the Gαq overexpression lysate to the untransfected lysate

Although an extensive examination of the physiological relevance and significance of GPCR priming is beyond the scope of this study, the ability of the non-cognate Gα peptide to stimulate signaling from cognate GPCR–G protein interactions leads to the testable prediction that increasing Gq interactions with β_2_-AR would lead to a corresponding increase in cAMP downstream of the Gs pathway. To retain the physiological context of priming, we stimulated endogenous β-ARs in HEK cells with increasing concentrations of fenoterol, synergized by treatment with forskolin (Fig. 7a and Supplementary Fig. 5). The concentrations of forskolin did not stimulate an increase in the cAMP response in the absence of the agonist (Supplementary Fig. 5). First, we monitored the effect of increasing non-cognate Gα subunit on cAMP by overexpressing Gαq protein (mCer-tag). Compared with an equivalent number of untransfected HEK cells, cells overexpressing Gαq (4- to 10-fold, Fig. 7c, d) had a significant increase in cAMP levels in response to fenoterol stimulation (Fig. 7a). Concentration–response curves for the transfected and untransfected cells start to diverge at sub-saturating concentration of fenoterol, but exhibit a similar potency (EC_50_ ~ 30 nM, Fig. 7a). At saturating fenoterol concentrations, Gαq-overexpressing cells have ~35% more cAMP than untransfected cells, suggesting that non-cognate expression levels can increase the magnitude of the cognate response. However, adenylate cyclase may be directly stimulated by PKCα and ζ^27^, which are known to operate downstream of Gαq. To test this confounding variable, PKC was inhibited by pretreatment of cells with 10 μM BimI or Gö6983^28,29^. Pretreatment with PKC inhibitors did not block the enhanced cAMP response from Gαq-overexpressing cells (Fig. 7b). These results demonstrate the interplay between cognate and non-cognate G proteins on canonical GPCR signaling in cells.

## Discussion

**Fig. 8:**
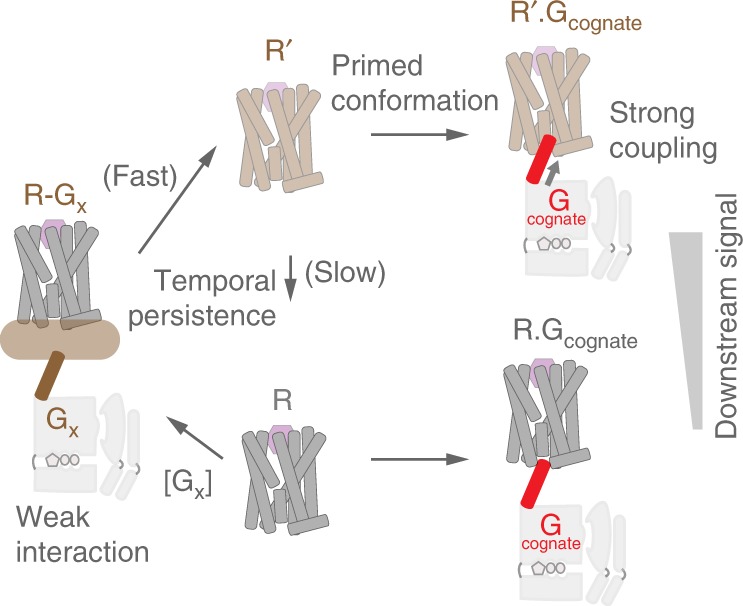
Temporal allosteric modulation of GPCR signaling. Agonist-bound GPCR (R) couples to a cognate G protein. Alternately, R interacts weakly with a G protein at either the canonical G-protein interface or a secondary binding site. Although this interaction does not trigger G-protein activation, it primes GPCR conformation (*R*’). *R*’ persists for several minutes before reverting to *R*. The primed conformation is characterized by stronger interactions with the cognate G protein leading to higher efficacy of G-protein activation and downstream signaling. The long-lived, higher efficacy *R*’ species creates a link between consecutive interactions at the cognate G-protein site resulting in temporal allosteric modulation of GPCR signaling

This study advances a molecular mechanism for the phenomenon of GPCR priming (Fig. 8), which denotes the synergistic effects of G protein interactions with a GPCR^9^. Our data show that interaction of both non-cognate and cognate Gα subunit C termini with the GPCR alter the receptor conformational state (Fig. 4). Following dissociation of the Gα C terminus (Fig. 3), the altered receptor conformational state has a half-life of 1½ min (Fig. 5), during which time the GPCR displays enhanced cognate G-protein activation (Figs. 4 and 6) and second messenger generation (Fig. 6). Delineating this molecular mechanism for GPCR priming reveals three aspects of GPCR signaling. First, GPCR–G protein interactions that do not precipitate G-protein activation are generally ignored as non-productive^30,31^. Instead, our study shows that non-productive GPCR–G protein interactions can influence signaling through their impact on receptor conformation. Consequently, varying the expression levels of non-cognate G proteins can modulate cognate signaling through endogenous receptors (Fig. 7). Second, the GPCR retains memory of previous interactions for an extended time (1½ min) leading to the temporal coupling of sequential GPCR–G protein interactions. Third, non-cognate GPCR–G protein interactions can increase coupling efficacy of cognate GPCR–G protein interactions. These three aspects of GPCR priming are consistent with the emerging view of receptors populating dynamic conformational landscapes^32–39^. Specifically, a combination of MS, nuclear magnetic resonance, and fluorescence spectroscopy studies have established that rather than functioning as on–off switches, GPCRs populate a broad conformational landscape, with ligand and G protein stabilizing one or more conformational sub-states. From this perspective, our data suggest that non-productive receptor–effector interactions can influence the GPCR conformational landscape, leading to higher efficacy of coupling with the cognate G protein.

Our study brings together a range of complementary experimental measurements that together support the temporal coupling of sequential GPCR–G protein interactions. (a) We had previously characterized three hotspot residues in the Gαs and Gαq C termini that mediate selective interactions with their respective cognate receptor-binding sites (β_2_-AR and V_1A_R)^17^. Mutagenesis of the cognate Gα C-terminal residues to their non-cognate counterparts enhanced GPCR priming, supporting a role for the canonical G-protein-binding site (Fig. 2). It must be noted that although these mutations are designed to impact canonical G-protein site interactions, they could also influence interactions at a secondary receptor-binding site. Regardless, binding of the non-cognate G protein to the receptor leads to priming (Fig. 8). (b) Both the cognate and non-cognate Gα C termini alter receptor conformation as measured by an intramolecular FRET sensor (Fig. 5b). The altered GPCR conformation persists for 1½ min following removal of the non-cognate Gα C terminus (Fig. 5d-f). (c) Both cognate and non-cognate Gα C termini enhance GPCR interaction with the cognate Gα C terminus, which is an established measure of the GPCR activation state (Fig. 3d). GPCR activation state persists for 5½ min following removal of the non-cognate Gα C terminus (Fig. 4d). The longer persistence of activation compared with receptor conformation (5½ vs. 1½ min; Supplementary Table 1) likely stems from the feedback on receptor conformation during sensor detection of activation state. Specifically, the SPASM sensor uses the cognate Gα C terminus to report on the GPCR activation state^14^. In turn, the GPCR-cognate Gα C terminus interaction prolongs the effects on receptor conformation. This feedback on receptor conformation is also consistent with the linear decrease in β_2_-AR-Sp response (Fig. 4d) rather an exponential decay, as observed in β_2_-AR-ICL3 (Fig. 5f), which would be expected as the receptor stochastically reverts from the primed conformation. (d) Presence of cognate and non-cognate Gα C termini enhance G-protein activation (Figs. 1 and 2). Enhanced G-protein activation persists for at least 1½ min following the removal of the non-cognate Gα C terminus (Fig. 6). (e) Lastly, the temporal coupling of sequential G protein interactions with a GPCR intertwines non-cognate and cognate signaling pathways, as witnessed by the influence of non-cognate expression levels on cognate signaling (Fig. 7).

We have rigorously investigated the validity of the temporal persistence observations. In particular, we sought to avoid artifacts stemming from the incomplete removal of the Gα C-terminal peptides in assays, where they were used as a G protein surrogate to prime the receptor. First, we used concentration–response curves to establish a critical or threshold concentration of the Gα C-terminal peptides to detect an enhanced GPCR activation state (Fig. 3d) using the SPASM sensor (Gαq—6.3 μM; Gαs—1.5 μM). Second, using quantitative immunoblottings and mass spectrometry, we confirmed that the non-cognate peptides were depleted below the critical concentration (residual peptide—affinity sequestration, 1.6 μM (Supplementary Fig. 2); photocleavage—4.58 μM (Supplementary Fig. 3)). Notably, these measurements of peptide depletion were performed in the presence of receptor membrane preparations to avoid shielding of the peptide by membrane/receptor interactions. Third, by monitoring the sequential pulldown and release of the receptor–peptide complex, we established that cognate Sp and non-cognate Qp exhibit predictable differences in the ability to interact with the receptor, although both exhibit weak (*K*_D_ ~ μM) interactions (Fig. 3c). The receptor dissociates from the Gα C-terminal peptide within 30 s (Fig. 3c). Fourth, we used a competition pulldown assay to demonstrate that the fragments produced by photocleavage of PC-Qp dissociate from the receptor within the time course of the UV exposure, and do not compete with Qp for interaction with the receptor (Fig. 3g). These data demonstrate that the temporal persistence of GPCR activation state (5½ min, Fig. 4), GPCR conformation (1½ min, Fig. 5), and G-protein activation ( > 1½ min, Fig. 6), established using orthogonal approaches (affinity sequestration and photocleavage), are not dependent on the sustained interaction of the receptor with the Gα C-terminal peptides. Further, the in vitro reconstitution of persistent G-protein activation using urea-stripped membranes overexpressing the receptor, recombinant G protein, and Gα C-terminal peptides is not consistent with a mechanism involving the post-translational modification of the receptor. Together, all our findings are consistent with the minute-scale persistence of receptor conformation state that augments downstream signaling.

One of the most intriguing findings of this study is the temporal persistence of a GPCR conformational state, and G-protein activation, for at least 1½ min following the removal of the non-cognate Gα C-terminal peptide. Within the GPCR signaling cascade, β-arrestin2 is reported to continue signaling from clathrin-coated structures for minutes, even after dissociation from β_1_-AR^13^. Beyond GPCR signaling, the phenomenon of temporal association between sequential interactions is well documented for enzymatic reactions. Turnover rates of single-enzyme molecules are influenced by previous catalytic cycles for cholesterol oxidase^40^, horseradish peroxidase^41,42^, β-galactosidase^43^, and an engineered lipase^44^. Underlying these observations is the concept of conformational or molecular memory in proteins, which indicates that subsequent steps in activity are influenced by the prior history of that molecule and not by the most recent/current state of the molecule alone. Molecular memory has also been observed for non-enzymatic proteins such as light-harvesting complex 2^45^, Grb2^46^, the ion channel hTREK1^47^, and the exchange factor Sos^48^. Molecular memory is also related to temporal allostery or allokairy, whereby the dynamic equilibrium between different activity states of an enzyme leads to the concentration-dependent temporal coupling of sequential enzyme–substrate interactions^49,50^. For GPCRs, we speculate that molecular memory stems from changes in transition rates that dictate interconversion between sub-states on the receptor’s dynamic conformational landscape.

The data presented here improve the mechanistic understanding of GPCR priming in context of ligand stimulation. However, our data cannot explain how priming can be both exceptionally long lasting and be subject to augmented signaling introduced by exogenous peptides or overexpressed G proteins. The persistence of the primed conformation suggests that receptors should be constantly primed in intact cells, which is not consistent with the classic equilibrium view of the Ternary Complex Model (Supplementary Figs. 6 and 7)^2^. Detailed kinetic analysis is necessary to better understand this phenomenon and its physiological implications. Structural details of the primed GPCR conformation and, in particular, its stability (1½ min) require additional investigations that are outside the scope of this study.

There is an increasing emphasis in structure-based drug discovery efforts to identify GPCR allosteric modulators that bind at sites distinct from the orthosteric ligand-binding pocket^51,52^. The benefits of allosteric modulators include the modulation of endogenous ligand signaling and the ability to harness unique structural motifs that allow distinction between closely related receptor isoforms^53^. In contrast, this study reveals the temporal allosteric modulation of receptors through sequential interactions at a single spatial binding site. Unlike traditional allosteric modulators that increase their effects (positive or negative modulation) in a concentration-dependent manner, our data show that temporal allosteric modulators (TAMs) such as the Gα C termini display an optimal concentration (Fig. 3d) that balances their positive effect on receptor conformation with the competitive inhibition of effector binding. Intriguingly, the molecular mechanisms of receptor signaling modulators, such as pepducins^54^, with the potential to bind at the cytosolic G-protein-binding pocket of GPCRs, remain unknown and need to be evaluated within the TAM framework.

## Methods

### Reagents

Ascorbic acid, isoproterenol ( + )-bitartrate salt, fenoterol hydrobromide, forskolin, and BimI were purchased from Sigma-Aldrich. Gö6983 was purchased from Selleck Chem. BODIPY-FL-GTPγS was from Life Technologies. Polyethyleneimine (PEI) 25 kDa linear polymer was purchased from Polysciences. Streptavidin-coated magnetic beads (Dynabeads) were purchased from New England Biolabs (catalog number S1420S). Complementary DNA encoding human β_2_-AR, human Gαq, and long splice variant of Gαs were obtained from Open Biosystems. Human V_1A_R cDNA was purchased from DNASU Plasmid Repository. Purified Gαq (*Mus musculus*), Gαs long (*Rattus norvegicus*), and Gβ_1_γ_2_ heterodimer were purchased as purified lyophilized proteins from Kerafast^55^.

### Constructs

All SPASM sensor constructs were expressed in pcDNA5/FRT (ThermoFisher). GPCR (β_2_-AR or V_1A_R), mCitrine (FRET acceptor), 10 nm ER/K α-helix, mCerulean (FRET donor), and Gα C terminus peptide were sequentially cloned using unique restriction sites. Control sensors had repeating (Gly-Ser-Gly)_4_ residues after mCerulean in place of the Gα C terminus peptide. A (Gly-Ser-Gly)_x4_ linker was inserted between all protein domains as part of the primer sequence to allow for free rotation between domains. An N-terminal hemagglutinin tag was inserted in-frame to all β_2_-AR sensors. Gα C terminus peptides encompassed the last 27 C-terminal residues of the corresponding Gα subunit^21^. Amino acid sequences used are listed in Supplementary Table 2.

Intramolecular FRET sensor (β_2_-AR-ICL3) was engineered on the basis of previous reports^25,26^. β_2_-AR-ICL3 was constructed from β_2_-AR (−) sensor by inserting an AgeI site between Asp-251 and Gly-252 in the third intracellular loop of β_2_-AR. Following restriction digestion, an mCerulean with (Gly-Ser-Gly)_x4_ linkers was ligated into the AgeI site in β_2_-AR. The C terminus of this sensor ended in mCitrine, similar to the β_2_-AR-(-) SPASM sensor. Primers used are listed in Supplementary Table 3. β_2_-AR-mCherry, β_2_-AR-mCerulean, and mCerulean-Gαq were constructed in a similar manner. All constructs were confirmed by sequencing.

### Synthetic peptides

Peptides corresponding to the α5 sequence from the C terminus of Gα proteins and peptides containing the hotspot mutations were chemically synthesized by GenScript at > 90% homogeneity. Peptide sequences are as mentioned above. Lyophilized peptides were dissolved in water and concentration determined by UV-absorbance at 280 nm. Biotinylated Qp was synthesized with an N-terminal biotin tag on Qp. PC Q-peptide was synthesized by replacing Y356 by 3-amino-3-(2-nitro)phenyl-propionic acid (Anp) in the Qp sequence to obtain PC-Qp: DTENIRFVFAAVKDTILQLNLKE(Anp)NLV and dissolved in dimethylsulfoxide. The vasopressin receptor agonist Arg8-vasopressin peptide was also purchased from GenScript.

### Cells, cell culture, transfection

HEK293 Flp-In T-Rex (hereafter HEK293T, ThermoFisher, catalog number R78007) cells were cultured in Dulbecco’s modified Eagle’s medium (DMEM) supplemented with 10% (v/v) fetal bovine serum (FBS), 4.5 g/L d-glucose, 1% GlutaMax, 20 mM HEPES, pH 7.5 at 37 °C. Cells were maintained in a humidified atmosphere with 5% CO_2_ and passaged regularly. For cAMP, IP_1_, and ΔFRET assays in cells, six-well tissue culture-treated plates were used. HEK293T cells (1 × 10^6^) were plated in each well and allowed to adhere for at least 16 h. Adhered cells were transfected with 1.5–2 μg DNA and 4.5–6 μl XtremeGENE HP DNA transfection reagent (Roche) mixed in Optimem (ThermoFisher). The length of transfection (20–28 h) was optimized to consistently yield equivalent levels of expression of all sensors in each experiment. Fresh medium was added to the cells on the day of the experiment, to prevent acidification-related stresses. For all experiments, sensor integrity, localization, and ratio of sensor expression to scattering (at 450 nm) were tracked to ensure consistency. Experiments were conducted at 60–80% transfection efficiency evaluated by ×20 and ×40 magnification on a Nikon TS100 microscope equipped with 100 W Hg-arc lamp and enabled with fluorescence detection. In addition, at the time of the experiment, 60–80% of transfected cells expressed predominantly plasma membrane-localized sensor with minimal localization to the intracellular compartments. Cells were resuspended by gentle pipetting in the culture medium for use in the assay.

For membrane preparations, 4 × 15 cm tissue culture-treated dishes were transfected at 50–60% confluence, using linear PEI. A 1 mg/ml solution of PEI was prepared in endotoxin-free water (ThermoFisher) heated to 80 °C. Upon cooling, the solution was neutralized to pH 7 with HCl. The solution was filter sterilized using a 0.22 μM syringe filter, aliquoted, and stored at −20 °C. Transfection mixtures were prepared in 500 μl Optimem by adding either 10 μg (β_2_-mCherry/β_2_-mCerulean/V_1A_R-mCherry) and 35 μl PEI solution or 20 μg (β_2_-AR-Sp) and 70 μl PEI solution. The solutions were pipetted gently and allowed to stand for 15–30 min before adding them onto a 15 cm dish each, with continuous swirling to ensure uniform exposure. Four hours after initiating transfection, the medium was replaced. Transfection was allowed to progress for 20 h (β_2_-mCherry/β_2_-mCerulean/V_1A_R-mCherry/β_2_-AR-ICL3) or 27 h (β_2_-AR-Sp) before collecting the cells for membrane preparations.

### Fluorescence measurement of sensor expression

Cells in each well from a six-well dish were resuspended by gentle pipetting in culture medium. The cell suspension was centrifuged at 300 × *g* for 3 min at ambient temperature and washed twice with phosphate-buffered saline (PBS) containing 0.02% glucose and 800 μM ascorbic acid to obtain 1 mL of single cells in suspension. One hundred microliters of this suspension was examined in an optical quartz cuvette (3–3.30-SOG-3, Starna Cells, Inc.) using a FluoroMax-4 fluorometer (Horiba Scientific). Exciting the cells at 430 nm (bandpass 8 nm) and scanning the emission from 450 to 600 nm (bandpass 4 nm) produced FRET spectra with distinct peaks at 475 nm (mCerulean) and 525 nm (FRET). Sensor expression was monitored from direct mCitrine fluorescence intensity (peak at 525 nm) obtained from an emission spectrum scanned from 500 to 600 nm (bandpass 4 nm) following excitation with 490 nm (bandpass 8 nm). Sensor integrity was determined from the ratio of mCitrine:mCerulean at their respective excitation and emission wavelengths. All experiments were conducted at mCitrine to mCerulean fluorescence emission ratios of 1.8–2.1, based on their respective excitations. Cells for membrane preparation were treated similarly and were resuspended in the same ratio of medium to buffer. The same O.D., mCitrine, and sensor integrity criteria were used.

### Membrane preparations

Membranes were prepared following a published protocol^56^ from HEK293T cells transiently transfected with the appropriate DNA (β_2_-AR-mCherry, β_2_-mCerulean, V_1A_R-mCherry, β_2_-AR-ICL3 or β_2_-AR-Sp). Cells were collected in culture medium and washed twice with PBS by centrifugation (300 × *g*, 3 min, ambient temperature). Cell pellets were incubated with an ice-cold hypotonic buffer (solution A: 10 mM HEPES, 1 mM EGTA, pH 7.2) for 30 min on ice. Cells in solution A were lysed gently (30 strokes) in a chilled Dounce homogenizer in the presence of 1.5 μg/mL aprotinin, 1.5 μg/mL leupeptin, 5 μg/mL phenylmethylsulfonyl fluoride (PMSF) and 1 mM dithiothreitol (DTT). Nuclei and intact cells were eliminated by centrifugation at 1000 × *g*, 5 min, 4 °C. All subsequent centrifugation steps were performed in a TLA100.4 rotor at 135,000 × *g*, 30 min, 4 °C. Native membranes were washed with 20 mM HEPES, 100 mM NaCl, 5 mM MgCl_2_, 100 μM GDP in the presence of 1.5 μg/mL aprotinin, 1.5 μg/mL leupeptin, 5 μg/mL PMSF, and 1 mM DTT, and were stored in the same buffer with 12% sucrose. For experiments to monitor Gα peptide–GPCR interactions using ΔFRET response, the native β_2_-AR-Sp sensor-containing membranes were washed in buffer containing 20 mM HEPES, 100 mM NaCl, 45 mM KCl, 5 mM MgCl_2_, and 100 μM GDP, and were stored in sucrose (12% w/v)-supplemented wash buffer.

For experiments in which activation of purified G proteins was measured, the membranes were treated with urea to denature and inactivate endogenous G proteins^56–59^. The membrane pellet was treated with 7 M urea in solution A for 30 min on ice. Urea was diluted to 3.5 M and membranes were pelleted. Urea-treated membrane pellet was resuspended in solution A containing 12% sucrose (w/v), aliquoted, frozen in liquid nitrogen, and stored at −80 °C. Total protein concentration (mg/mL) was calculated using a DC Protein Assay (Bio-Rad).

### ΔFRET assay on membranes

Native membranes were collected from HEK293T cells expressing either the β_2_-AR-Sp SPASM sensor or the β_2_-AR-ICL3 conformational sensor. Frozen membranes were thawed and handled on ice. The membranes were resuspended by sonication to a concentration of 100 nM sensor (1 × 10^6^ mCer cps) in 20 mM HEPES, 100 mM NaCl, 45 mM KCl, 5 mM MgCl_2_, pH 7.4 (ΔFRET buffer minus Ca^2+^) in the presence of 1.5 μg/mL aprotinin, 1.5 μg/mL leupeptin, and 5 μg/mL PMSF. Indicated peptides were added to 30 μM (50 μM for PC-Qp) in a sample volume of 1 mL. The sample was aliquoted as 90 μl into ten replicate tubes—five labeled ISO and five buffer. Agonist solution (ISO) containing 1 mM Isoproterenol and 1 mM ascorbic acid was prepared in ΔFRET buffer minus Ca^2+^. A buffer control consisting of 1 mM ascorbic acid in the same buffer was used as stimulation and dilution control. With a count-down timer set to 11 min, 10 μl of ISO were added (stimulation) to the appropriate sample tube at every 1 min interval. The sample was mixed by pipetting and incubated at 25 °C for 5 min 300 r.p.m. in a block shaker, prior to acquisition of the FRET spectrum. FRET emission spectra were acquired on 90 μl of the aliquot in an optical quartz cuvette (3–3.30-SOG-3, Starna Cells, Inc.) using a FluoroMax-4 fluorometer (Horiba Scientific). Exciting the sample at 430 nm (bandpass 8 nm) and scanning the emission from 450 to 600 nm (bandpass 4 nm) produced FRET spectra with distinct peaks at 475 nm (mCerulean, donor) and 525 nm (mCitrine, FRET acceptor). The ratio of emission intensities at 525 nm amd 475 nm after background correction is the FRET ratio (ISO). The procedure (stimulation, mixing, incubation, FRET spectrum acquisition) was repeated for control samples that received 10 μl buffer each, to yield FRET ratio (buffer).

1 $$\Delta FRET\,ratio = FRET\,ratio\left( {ISO} \right) - FRET\,ratio(buffer)$$

For the concentration–response curves monitoring ΔFRET as a function of peptide concentration, 17 working samples of 1 ml volume were prepared from the same batch of β2-AR-Sp sensor membranes for one experiment. Eight of these were treated with Sp, eight with Qp, and one was a test of the membrane response in the absence of peptide. Each tube was treated with one peptide concentration in the range of 100 nM to 100 μM. Each working sample was aliquoted as 90 μl × 10 tubes, and processed as above to obtain ΔFRET ratio. Values were fit to a biphasic response and first-order derivative of the fit was used to determine the critical concentration (*C*_*C*_).

In the experiments designed to test temporal persistence using depletion of bioQp, the no-bead samples were measured as in the standard ΔFRET assay. The Beads-treated samples were stimulated and incubated for 3 min. They were subsequently treated with 20 μl of 0.4 mg/ml streptavidin-coated magnetic beads and resuspended in ΔFRET buffer minus Ca^2+^. After mixing, beads were pulled down using a Neodymium disc magnet N52 (20 × 40 mm). Supernatant was pipetted into a quartz cuvette for the rest of the incubation (1½ min) and FRET spectrum was acquired on the sample (5 min after stimulation was initiated). BioQp was preadsorbed/pre-depleted by incubating a 300 μM solution of the peptide with the appropriate volume of 0.4 mg/ml suspension of streptavidin-coated magnetic beads for 30 s. Beads were precipitated using the magnet and the supernatant was used to obtain FRET spectra and calculate the ΔFRET ratio.

In the experiments designed to test temporal persistence using depletion of PC-Qp, no UV samples were acquired as described. For the Pre-cleaved samples, a solution of each peptide was UV irradiated at 350 nm (bandpass 10 nm) for 2 min in a large quartz cuvette before being added to the membranes. For the samples labeled Cleavage 1 min post-mixing, the stimulation (ISO/buffer) was initiated at 25 °C, with 300 r.p.m. mixing. One minute later, UV irradiation was performed (350 nm, bandpass 10 nm, 2 min) in a quartz cuvette. The sample was further incubated for 2 min at 25 °C, with 300 r.p.m. mixing and then FRET spectra were acquired.

For monitoring ΔFRET over time, 500 μl membrane samples were stimulated (ISO/buffer) and incubated at 25 °C, with 300 r.p.m. mixing. The sample was either treated with the appropriate amount of beads (3 min after stimulation) for 30 s to deplete the bioQP or with UV radiation (1 min after stimulation) for 2 min to cleave the PC-Qp. The supernatant or irradiated sample was incubated at 25 °C, with 300 r.p.m. mixing. At the indicated time, 90 μl of sample was pipetted into a 3 mm quartz cuvette for acquisition of the FRET spectrum.

### In vitro reconstitution of G-protein activation

HEK293T cells in 15 cm dishes were transiently transfected with β_2_-AR-mCherry or V_1A_R-mCherry plasmid as described in the section on “Cells, Cell culture and Transfections,” earlier. Membranes were isolated and treated with urea as detailed (Membrane preparations). Urea treatment denatures and inactivates the endogenous G proteins, allowing the reconstitution of desired combinations of Gα, and βγ subunits and specific C terminus peptides. Reconstitution reactions were assembled on ice. They contained 60 μg of membrane in 20 mM HEPES, 100 mM NaCl, 5 mM MgCl_2_, 10 μM GDP, 0.1 mg/mL bovine serum albumin (BSA) and 1 mM DTT in a total volume of 600 μl. Indicated Gα and β1γ2-subunit were added to a final concentration of 100 nM. Soluble Gα C terminus peptides were added to achieve a concentration of 10 μM. A stimulation master mix containing 10 mM BODIPY-FL-GTPγS and either 100 μM fenoterol hydrobromide (for β_2_-AR membranes) or 1 μM vasopressin was prepared in PBS, containing 10 mM ascorbic acid. A mock solution containing identical buffer and 10 mM BODIPY-FL-GTPγS, but without agonist was also prepared. Both solutions were stored on ice. Ninety microliters of the reaction mixture was aliquoted into a 3 mm quartz cuvette. Ten microliters of stimulation buffer was added and mixed with the sample to obtain a final concentration of 1 mM BODIPY and either 10 μM fenoterol or 100 nM vasopressin. Control reactions were spiked with BODIPY-containing buffer, without agonist. BODIPY fluorescence (Early stimulation) was recorded within 15 s of sample stimulation with an excitation wavelength of 470 nm (bandpass 2 nm), collecting the emission spectrum from 485 nm to 600 nm (bandpass 4 nm). The spectrum exhibited a distinct BODIPY peak with maximum intensity at 511 nm, which was used for calculation. The sample cuvette was incubated at 37 °C for 3 min and a post-stimulation BODIPY spectrum was acquired. The agonist-stimulated change in fluorescence counts was calculated by subtracting the early stimulation spectra from the post-stimulation spectra. Samples treated with BODIPY buffer (no agonist) were processed similarly, to determine the increase in BODIPY fluorescence without agonist stimulation, which represented nonspecific and basal incorporation of the BODIPY in the reaction time. Specific, agonist-induced change in BODIPY fluorescence was calculated by subtracting the increase in buffer-treated samples from the agonist-mediated increase. This change in fluorescence (increase) provided a measure of BODIPY-FL-GTPγS incorporation into the Gα-subunit. Each reaction provided three agonist and three mock readings. Experiments were repeated with at least three independent membrane preparations. Experiments to study activation of endogenous G protein were performed by using native membranes and by following the same protocol.

A variation of this protocol was used for monitoring temporal persistence of G-protein activation after photocleavage of the non-cognate peptide. For positive controls, native membranes collected from β_2_-AR-mCherry cells were incubated in the same buffer as above with either no peptide, 30 μM Qp, or 50 μM PC-Qp. Agonist-stimulated and mock-treated spectra were acquired as above, to calculate the agonist-specific G-protein activation. For negative controls, a solution of either Qp (3 mM) or PC-Qp (5 mM) was irradiated in a quartz cuvette with 350 nm (bandpass 5 nm) for 50 s. Native membranes were mixed with either irradiated peptide 30 μM Qp or irradiated 50 μM PC-Qp. Agonist-stimulated and mock-treated spectra were acquired as above, to determine the effect of photocleavage on G-protein activation. Further controls involved irradiation of the stimulation buffer (PBS, ascorbic acid, fenoterol, BODIPY) and mock buffer (PBS, ascorbic acid, BODIPY) in addition to peptide irradiation. The irradiated peptide and buffer were mixed with native membranes and spectral acquisitions repeated to determine the effect of UV on the reactants. In the test condition, native membrane and either 30 μM Qp or 50 μM PC-Qp were incubated together in the same buffer as above. The reaction was stimulated with fenoterol in PBS containing 10 mM ascorbic acid. BODIPY incorporation was monitored as above. Prior to irradiation, the early-stimulation BODIPY spectrum was acquired. The reaction was irradiated with UV 1 min after the mixing was initiated. At 3 min after mixing, the post-stimulation BODIPY spectrum was acquired. The experiment was repeated with mock buffer and agonist-specific G-protein activation was calculated.

### cAMP measurements

HEK293T were transiently transfected with sensors using XtremeGENE HP (Roche) according to the manufacturer’s instructions. Twenty to 28 h post transfection, cells were gently resuspended in DMEM containing 10% FBS (v/v), centrifuged, and resuspended in PBS with 0.02% glucose and 800 μM ascorbic acid to a density of 4 × 10^6^ cells/mL as measured by a Countess II cell counter (ThermoFisher), and equivalent sensor expression as determined by fluorescence spectra (as described under “Fluorescence measurement of sensor expression”). For the β_2_-AR-ICL3 sensor, the cells were transfected and collected to obtain a range of sensor expression values as determined by fluorescence measurements. Cells (4 × 10^4^) were aliquoted into 96-well U-bottomed, opaque, white microplates (Corning). Cells were treated with 0.3 nM to 10 μM of one of three agonists (isoproterenol, fenoterol, or epinephrine) or 10 μM forskolin, or with no stimulation for 5 min at 37 °C. After stimulation, cells were processed for the cAMP Glo assay as per the manufacturer’s instructions (Promega). Luminescence was measured using a microplate reader (FlexStation3, Molecular Devices). The luminescence signal of stimulated cells was subtracted from that of cells exposed to buffer. Only those experiments in which the cAMP response of all sensors to isoproterenol was in the range of 50–70% of the response to forskolin, based on fits to the operational model performed earlier, were considered^9,60^.

For concentration–response curves with Gαq overexpression as well as the knockdown experiments, transfected and control cells (untransfected or mock short hairpin RNA transfected) were resuspended to equivalent concentration (4 × 10^6^ cells/ml) based on Countess II numbers. Stimulation was performed with 0.3 nM to 100 μM fenoterol in the presence of 2 μM forskolin for 10 min. As the response was being monitored from endogenously expressed β_2_-AR, it had a low magnitude and was synergized by the addition of forskolin^61^. The concentration of forskolin used did not produce a significant response in the absence of fenoterol (Supplementary Fig. 5). Effect of PKC inhibition on cAMP response was monitored by pre-treating the cells with 1 μM BimI or 1 μM Gö6983 for 15 min at 37 °C, and then stimulating them with 10 μM fenoterol and 2 μM forskolin for 10 min.

Data were normalized either to the maximum response from β_2_-AR-(-) sensor or to the maximum value observed from untransfected cells stimulated with fenoterol and forskolin. Concentration–response curves were fit to the form below to estimate EC_50_ and *E*_max_.

2 $$Y = E_{min} + \frac{{(E_{max} - E_{min})}}{{1 + 10^{\left( {logEC_{50} - x} \right)Slope}}}$$

where *E*_min_ is the cAMP response at the lowest concentration of fenoterol and *Slope* indicates the Hill coefficient of the isotherm.

### IP_1_ assay

HEK293T cells expressing the indicated sensor were collected 28–32 h post transfection, to assess IP_1_ levels using the IP-One HTRF assay kit (Cisbio). Cells were gently resuspended in their original media, counted using a hemocytometer, and centrifuged (300 × *g*, 3 min). An appropriate volume of StimB buffer (CisBio: 10 mM Hepes, 1 mM CaCl_2_, 0.5 mM MgCl_2_, 4.2 mM KCl, 146 mM NaCl, 5.5 mM glucose, 50 mM LiCl, pH 7.4) was added to reach a density of 3 × 10^6^ cells/mL. Cells were incubated at 37 °C for 15 min. Cells were subsequently incubated with or without 100 nM of Arg-vasopressin peptide for 5 min at 37 °C. The manufacturer’s protocol was modified to achieve a high signal-to-noise ratio. One hundred and fifty microliters of suspension was incubated on a shaker with 30 μl of lysis buffer (Cisbio), 54 μl StimB buffer, 6 μl IP_1_ conjugated to D2 dye, and 6 μl terbium cryptate-labeled anti-IP_1_ monoclonal antibody in a 384-well plate for 2 h. IP_1_ FRET spectra were collected in top read format using a FlexStation3 plate reader with a delay of 50 μs and an integration time of 300 μs. Excitation, emission, and cutoff wavelengths were 340, 665, and 630 nm (acceptor d2), and 343, 620, and 570 nm (terbium cryptate donor), respectively. FRET was calculated from ratio of the emission at 655 nm to the emission at 620 nm. Data are presented as a change in raw IP_1_ ratio following drug treatment. Each experiment had four repeats per condition and was repeated at least three times.

### Binding-and-release assay for Gα peptide–β_2_-AR interaction

Native membranes were prepared from cells expressing β_2_-mCer and stored in sucrose-supplemented buffer A as described above (Membrane preparations). Membranes were sonicated (3× bursts, 15 s with 1 min pauses) and resuspended in ΔFRET buffer minus Ca^2+^ to a concentration of 10 nM receptor based on fluorescence counts. bioQp/bioSp (30 μM) and 100 μM isoproterenol were added and mixed for 5 min. Samples were split into duplicates, one of which was not manipulated in any way to obtain total receptor counts. To the other tube, 20 μl of 0.4 mg/ml streptavidin-coated magnetic beads were added, mixed, and precipitated using a Neodymium disc magnet N52 (20 × 40 mm). Supernatant was removed to a fresh micro-centrifuge tube to monitor fluorescence of receptors not bound to the beads. A solution of 100 μM Qp/Sp was added to the precipitated beads, mixed, and magnetically separated again. The supernatant was collected to determine the released fraction of receptors. Fluorescence of mCer was monitored on a FluoroMax 4 fluorometer (Horiba) using 430 nm excitation (8 nm bandpass) and emission scan from 450 to 500 nm, with the maximum at 475 nm being used for the calculation. Based on total and unbound counts, the amount of receptor bound was calculated (bound = total − unbound) and compared with the counts from the released receptor (% = released/bound). To estimate the nonspecific interaction, the bead-mediated receptor pulldown was performed without biotinylated Gα peptide. *K*_D_ was estimated from the single point measurement of bound and unbound receptor concentration using the formula

3 $$K_D = \frac{{\left[ {total\,peptide} \right][free\,receptor]}}{{[peptide - receptor\,complex(specific)]}}$$

### Western blotting

Briefly, lysates were separated on 10% polyacrylamide/SDS gels and transferred to polyvinylidene difluoride (PVDF) membranes for 3 h at 300 mA. Blots were blocked with 5% milk/TBST for 1 h. Primary Gq antibody (1:1000, Life Technologies, PA5–79318), β_2_-AR (1:100, Abcam, ab61778), Gαs c-tail (1:750, Millipore, 06–237), or vinculin antibody (1:2000, Life Technologies, 700062) were used at the indicated concentrations in 1% milk/TBST for 1 h at room temperature. To minimize sample-loading errors that might confound interpretation of the loading control (vinculin), the same blot was split into top half (for vinculin) and bottom half (for Gαq, Gαs, β_2_-AR). To conserve the antibody reagent, indicated molecular-weight markers in the blotting were used to cut out and probe portions of the blotting with the specific antibodies. The protein bands appeared in the appropriate places as confirmed by the shift in size of the mCer-tagged Gαq. As the experiment deals with this overexpressed protein, which is clearly detectable, any nonspecific interactions do not impact the interpretation of data. Blottings were washed with TBST (3 × 10 min) before the addition of secondary antibody (goat anti-rabbit (Jackson ImmunoResearch Laboratories), 1:7500 (Gq), 1:5000 (β_2_-AR, Gαs c-tail), or 1:10,000 (vinculin) in 1% milk/TBST) and incubated at room temperature for 1 h. Blottings were washed again with TBST (3 × 10 min) and developed using Immobilon Western Chemiluminescent HRP substrate (Millipore). Blottings were imaged using an Odyssey system (Li-Cor Biosciences). Blotting images were prepared and analyzed using ImageJ/FIJI (NIH)^62,63^.

### Estimating depletion of bioQp

Native membranes were prepared from cells expressing β_2_-mCer and were stored in sucrose-supplemented buffer A as described above (Membrane preparations). Membranes were sonicated (3× bursts, 15 s with 1 min pauses) and resuspended in ΔFRET buffer minus Ca^2+^ to a concentration of 10 nM receptor into triplicate tubes. bioQp (30 μM) and 100 μM isoproterenol were added to two tubes and were mixed for 5 min. The third tube received isoproterenol alone. Serial dilutions of the sample containing 6, 3, and 1.5 μM of bioQp were prepared from one tube. The second tube was treated with 20 μl of 0.4 mg/ml streptavidin-coated magnetic beads. Beads were mixed and pulled down using the N52 magnet. Supernatants were collected and stored on ice. The samples were analyzed by slot blot as detailed below.

### Slot blots

Samples were resuspended in 1% Triton-X-100 and sonicated using a probe sonicator on ice (10× bursts 10 s with 15 s pauses). Samples were diluted to 10 nM β_2_-mCer based on fluorescence counts. Diluted samples were then passed through a Bio-dot SF slot blotter (Bio-rad) under vacuum onto PVDF (Hybond P, 0.2 μm pore size; Amersham) and were washed twice with equal volumes of the ΔFRET buffer minus Ca^2+^. Blottings were then blocked with 5% BSA/TBST for 1 h at room temperature, treated with 1:20,000 streptavidin–HRP (21130, Pierce) in 5% BSA/TBST for 1 h at room temperature, washed 3 × 10 min with TBST, and developed and analyzed as described above for western blottings.

### Estimating the extent of PC-Qp photocleavage

Native membranes were prepared from cells expressing β_2_-mCer and were stored in sucrose-supplemented buffer A as described above (Membrane preparations). Membranes were sonicated (3× bursts, 15 s with 1 min pauses) and resuspended in ΔFRET buffer minus Ca^2+^ to a concentration of 10 nM receptor into duplicate tubes. bioQp (50 μM) and 100 μM isoproterenol were added to two tubes and were mixed for 5 min. Sample from one tube was exposed to UV irradiation (350 nm, 10 nm bandpass, 2 min) in a quartz cuvette using a FluoroMax 4 fluorimeter. Both samples were centrifuged 100,000 × g, 5 min, 4 °C to sediment the membrane. Supernatants were stored on ice for analysis by MS. Control samples containing 50 μM peptide in the buffer were also prepared.

### Estimating the release of PC-Qp fragments from membrane/β_2_-mCer

Native membranes were prepared from cells expressing β_2_-mCer and were stored in sucrose-supplemented buffer A as described above (Membrane preparations). Membranes were sonicated (3× bursts, 15 s with 1 min pauses) and resuspended in ΔFRET buffer minus Ca^2+^ to a concentration of 10 nM receptor based on fluorescence counts. bioQp (30 μM)/30 μM bioQp + 30 μM Qp/30 μM bioQp + 50 μM Qp, and 100 μM isoproterenol were added and mixed for 5 min. Samples were split into duplicates, one of which was not manipulated in any way to obtain the total receptor counts. To the other tube, 20 μl of 0.4 mg/ml streptavidin-coated magnetic beads were added, mixed, and precipitated using a Neodymium disc magnet N52 (20 × 40 mm). Supernatant was removed to a fresh micro-centrifuge tube to monitor fluorescence of receptors not bound to the beads. Percentage of receptors bound to the beads was calculated by subtracting the unbound from the total (duplicate). The experiment was repeated with UV exposure for 2 min, followed by the addition and pulldown using the magnetic beads.

### Mass spectrometry

Prior to matrix-assisted laser desorption and ionization time-of-flight MS (MALDI-TOF MS) analysis, the samples were desalted using C18 ZipTips (Millipore Corporation, Billerica, MA). The manufacturer’s standard protocol for peptide clean-up was followed. After elution from the ZipTip, 1 μL of each sample was spotted on a MALDI-TOF 384 spot stainless-steel target (Bruker Daltonics, Billerica, MA) and mixed with 1 μl of α-cyano-4-hydroxy-cinnamic acid (α-CHCA) matrix (15 mg/ml α-CHCA in 50% acetonitrile). Each spot was allowed to dry at room temperature. The target was placed in a Bruker Autoflex Speed MALDI-TOF mass spectrometer (Bruker Daltonics, Billerica, MA) equipped with a Nd-YAG 355 nm pulsed laser. The data were collected in reflectron mode, positive polarity, with an accelerating potential of 19 kV. Each spectrum was the accumulation of 4000 laser shots with complete spot raster of the laser during data collection. External calibration was performed using 0.5 μl α-CHCA and 0.5 μl of peptide standard (part# 206195, Bruker Daltonics, Billerica, MA) with Angiotensin II (monoisotopic mass [MH^+^ ] 1046.5418), Angiotensin I (monoisotopic mass [MH^+^ ] 1296.6848), SubstanceP (monoisotopic mass [MH^+^] 1347.7354), Bombesin (monoisotopic mass [MH^+^] 1619.8223), ACTH clip 1–17 (monoisotopic mass [MH^+^] 2093.0862), ACTH clip 18–39 (monoisotopic mass [MH^+^] 2465.1983), and Somatostatin 28 (monoisotopic mass [MH^+^] 3147.4710). The Bruker data files were converted to mzXML files using MSConvertGUI tool in ProteoWizard open-source software^64^. The mzXML files were viewed and analyzed with mMass v5.5 open-source software^65^.

### Reporting summary

Further information on research design is available in the Nature Research Reporting Summary linked to this article.

## Supplementary information

Supplementary Information

Reporting Summary

Source Data

## Data Availability

Data supporting the findings of this manuscript are available from the corresponding author upon reasonable request. A reporting summary for this study is available as a Supplementary Information file. All data generated or analyzed during this study are included in this published article. The source data underlying Fig. 1b–i, 2b–d, 3c, d, e, g, 4c–e, 5b, d–f, 6b, c, 7a, b, d and Supplementary Figs. 1a–d, 2a, b, 3b, c, 4b, c, and 5 are provided as a Source Data file.
