# Supplementary material for: Minute-scale persistence of a GPCR conformation state triggered by non-cognate G protein interactions primes signaling
Source: Nat Commun. 2019 Oct 23;10:4836. doi: 10.1038/s41467-019-12755-9 (PMC6811539; doi:10.1038/s41467-019-12755-9)
Supplement: Supplementary file 1 — Supplementary Information [file 41467_2019_12755_MOESM1_ESM.pdf]

Supplementary Information

Supplementary information for

Minute-scale persistence of a GPCR conformation state triggered by non-cognate G protein interactions primes signaling.

Gupte et al.

Contents-

Supplementary Figure 1 – Supplementary Figure 7

Supplementary Table 1 – Supplementary Table 3.

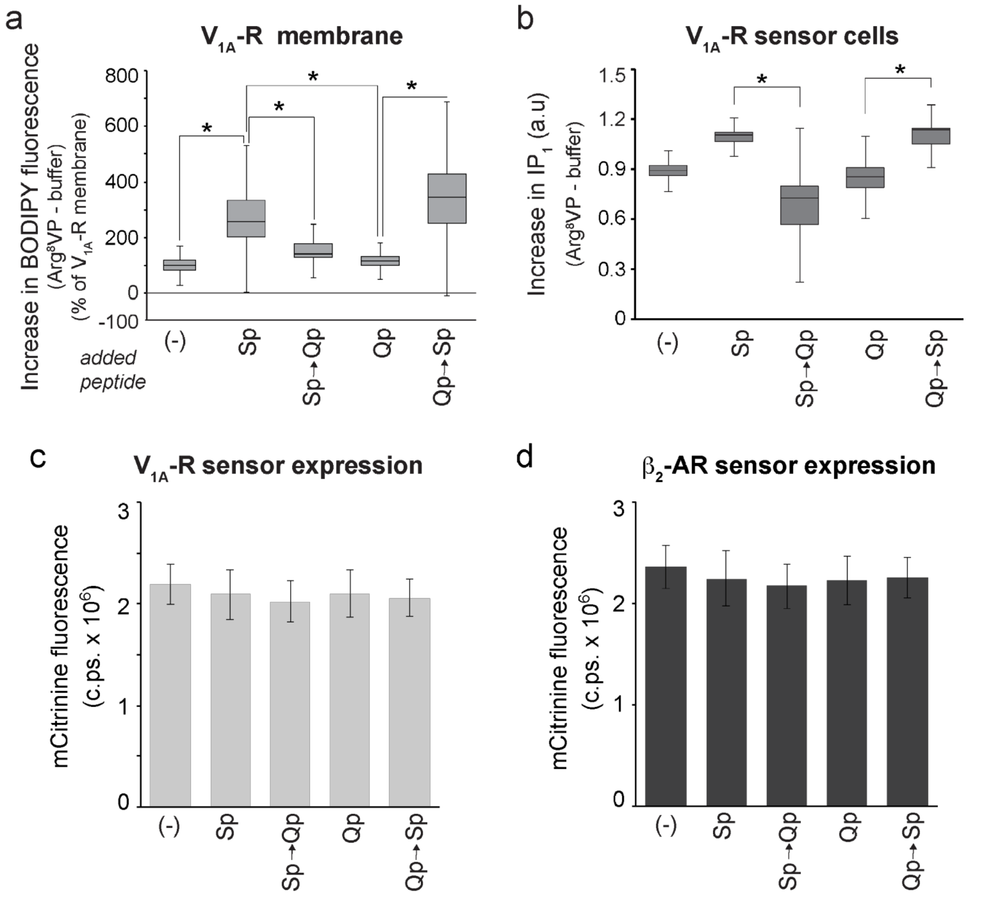

Supplementary Figure 1 - Hot-spot substitutions in Gα peptides affect G protein activation and downstream signaling from V_{1A}-R. (a) Activation of G protein (Gαq) is monitored by the increase in BODIPY fluorescence upon agonist stimulation of membranes harvested from HEK293T cells expressing V_{1A}- R, and reconstituted with purified Gαq. Presence of the indicated Gα peptides, (cognate (Qp), non- cognate (Sp), cognate to non-cognate (Qp → Sp), and non-cognate to cognate (Sp → Qp)) affects the magnitude of G protein activation, expressed as a percentage normalized to the change observed in the absence of added peptide (-). (b) Downstream signaling from cells expressing equivalent amounts of V_{1A}-R SPASM sensor was measured by monitoring IP_1 levels following agonist stimulation. Box-and-whisker plots: centre line is median, box ends are upper and lower quartiles, whisker ends are *1.5×interquartile range (IQR)* from four independent experiments with at least three replicates per experiment (*$n \geq 4$*). Statistically significant differences were assessed by a one- way ANOVA, followed by Tukey's post-ANOVA test. Significance is denoted by asterisks, *, *$p<0.05$*.

Equivalent sensor expression of the different V_{1A}-R SPASM sensors (c) and β_2-AR SPASM sensors (corresponding to downstream assays in Fig. 2d) is inferred from the fluorescence intensity of equal numbers of cells for each sensor. Values are mean ± S.D from at least 3 separate experiments.

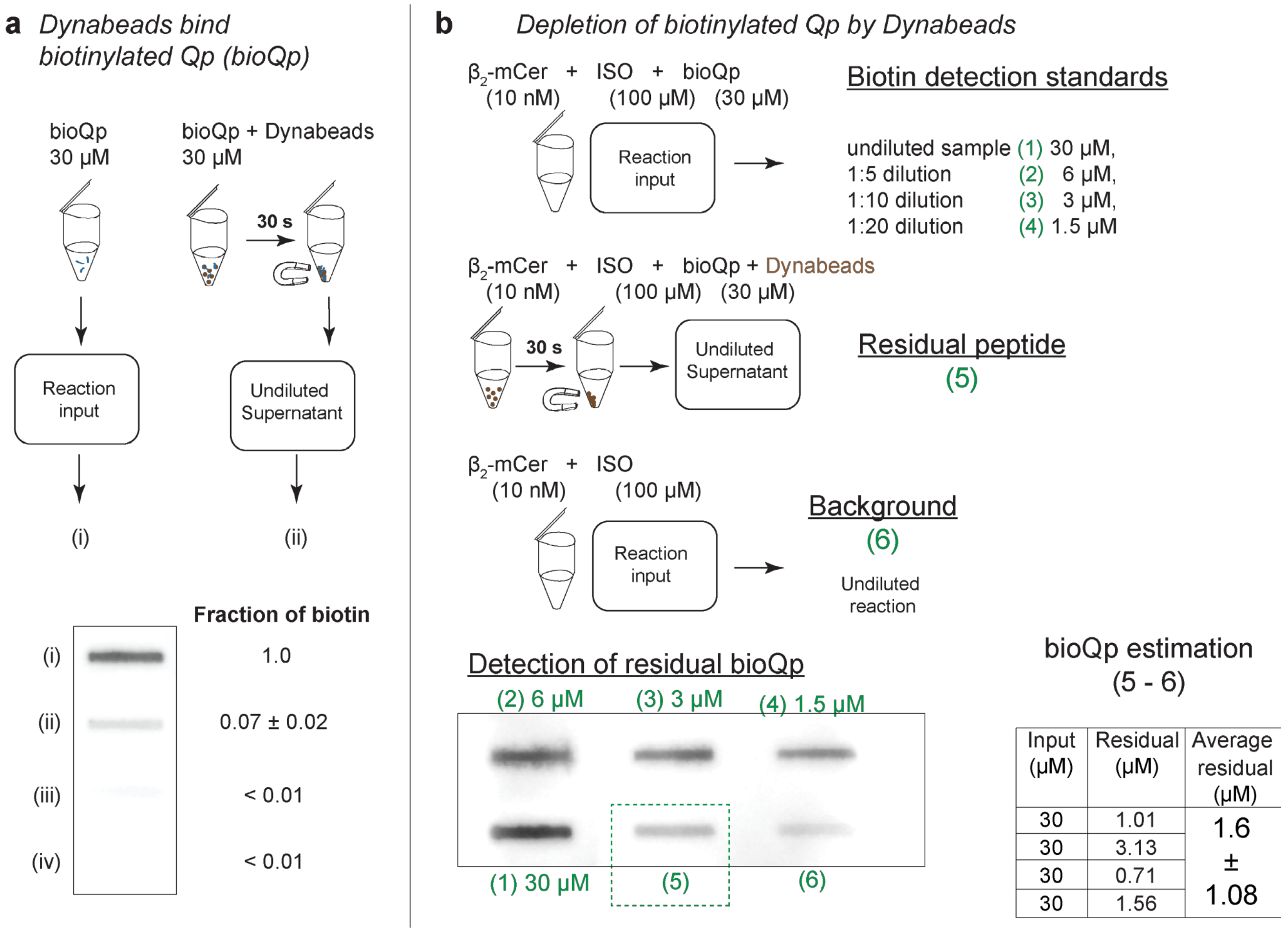

Supplementary Figure 2 - Depletion of biotinylated Qp. (a) Streptavidin-coated magnetic beads (brown circles) bind to biotinylated Qp (bioQp) from solution. Binding is quantified by Western blotting of the samples for biotin (i- 30 µM bioQp, ii- supernatant after binding to Streptavidin-coated magnetic beads, iii - 30 µM Qp, iv- buffer control). (b) Streptavidin-coated magnetic beads (brown circles) deplete bioQp to sub-critical concentration, in the presence of β_2-mCer membranes. Reactions containing 30 µM bioQp, 10 nM β_2-mCer and isoproterenol were probed by immunoblotting at the indicated dilutions (reflecting theoretical bioQp concentration (1-4)). These were compared to the undiluted supernatant obtained after treatment of the sample with streptavidin-coated magnetic beads for 30 s (5), and a no peptide control (6). Treatment of the samples with Streptavidin-coated magnetic beads reduces bioQp concentration to 1.6 µM across 4 experiments.

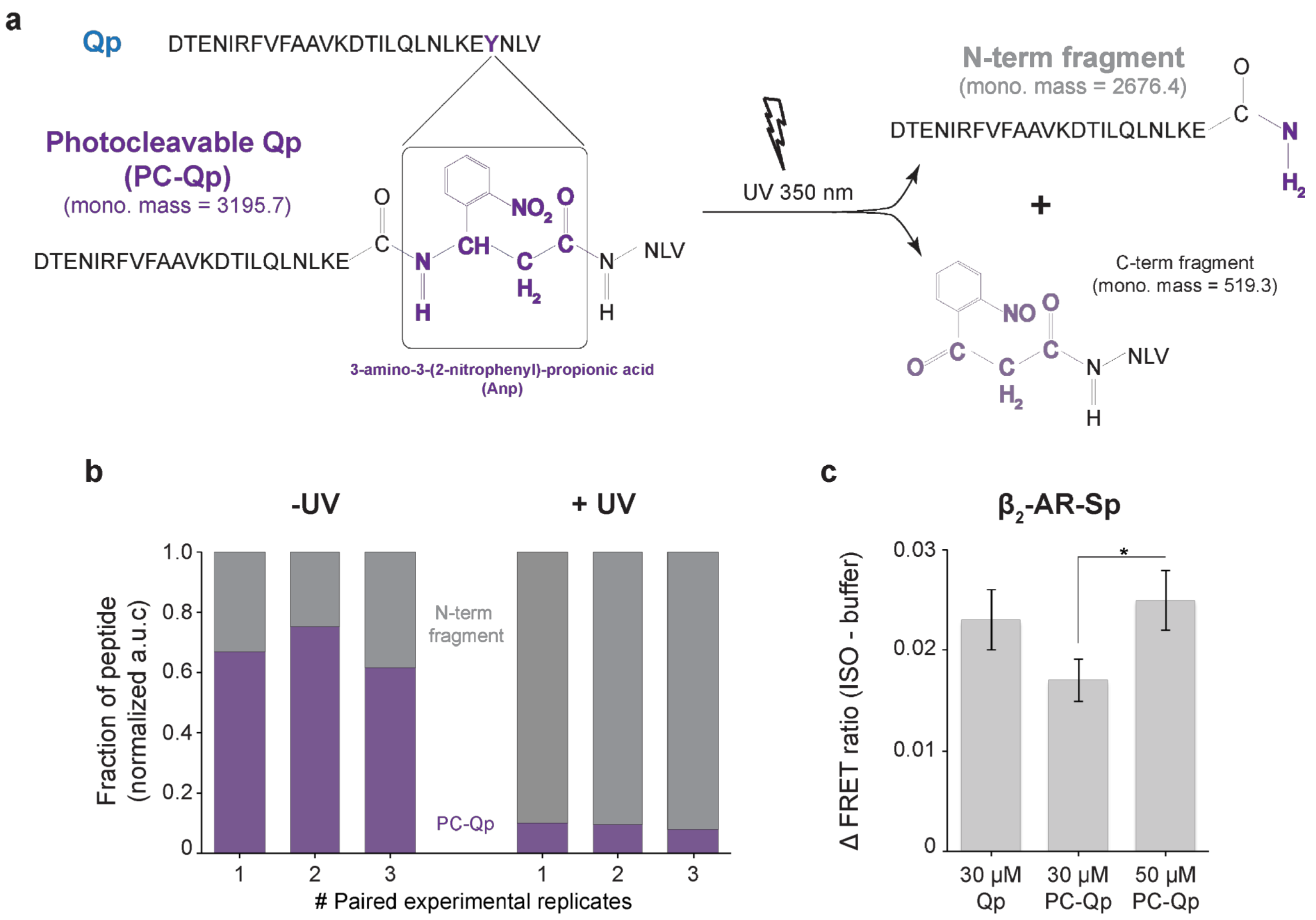

Supplementary Figure 3 - Photocleavage of PC-Qp by UV irradiation. (a) Design of photocleavable Qp (PC-Qp) by replacing the indicated Tyrosine (Y356) residue in Qp sequence with 3-amino-3-(2-nitro)phenyl- propionic acid (Anp). Reaction scheme depicting photocleavage products of PC-Qp upon UV irradiation, with average m/z of the resulting fragments. (b) Quantification of the extent of photocleavage of PC-Qp by MALDI-MS. UV irradiation of full length PC-Qp in the presence of native membranes causes the concentration of the full length PC-Qp to fall to 4.58 µM, which is less than the critical concentration (C_C) required for GPCR priming. (c) Change in FRET ratio following isoproterenol treatment (100 µM) of β_2-AR-Sp sensor in the presence of indicated concentrations of Qp or PC-Qp. Values are mean ± S.D from 3 experiments (b) or mean ± S.D from at least 12 replicates across 3 experiments (c). Statistically significant differences were assessed Students' *t*-test test. Significance is denoted by asterisks, *, *p<0.05.*

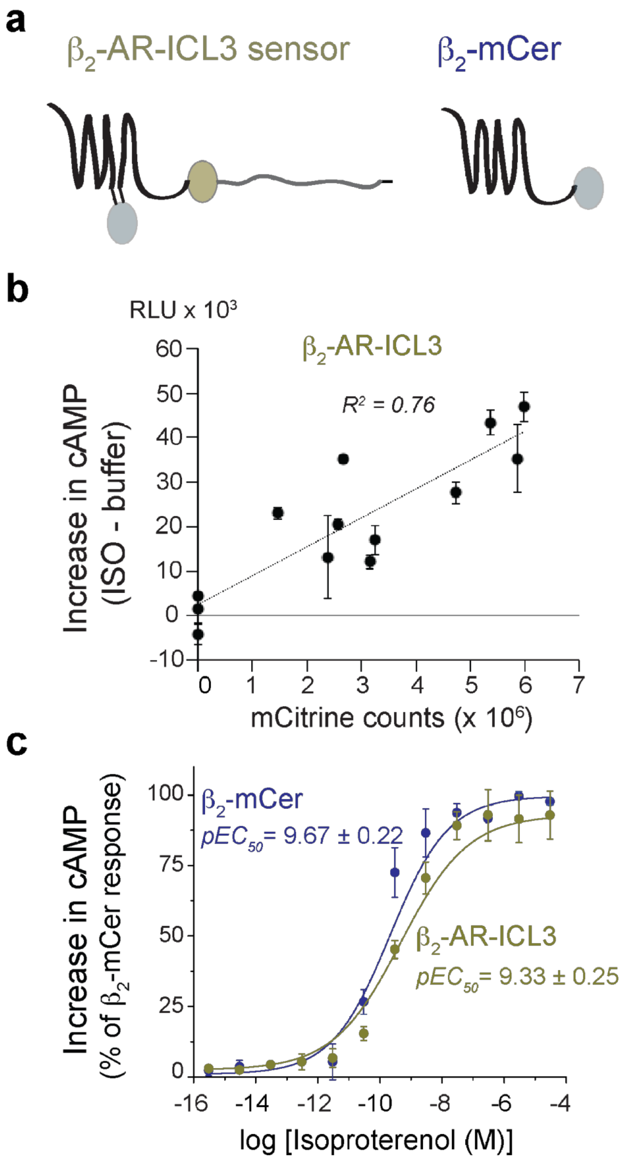

Supplementary Figure 4 – β2-AR-ICL3 is capable of stimulating downstream signaling. (a) Schematic representation of the β2-AR-ICL3 sensor designed to report on conformational changes in the receptor, compared to the wild-type β2-AR-mCer. (b) cAMP accumulation in HEK293T cells expressing increasing amounts of β2-AR-ICL3 sensor, upon stimulation with isoproterenol (10 μM), compared to buffer-treated cells. Increasing sensor expression correlates with greater cAMP accumulation ($R^2 = 0.76$). Each data point represents mean ± S.D from 4 replicates in a single experiment. Experiments were repeated thrice to obtain multiple x-y pairs. Data was fit to a linear regression to find correlation coefficient. (c) Cells expressing either the control β2-AR-mCer or the β2-AR-ICL3 sensor to equivalent levels were stimulated with varying concentrations (0.3 fM to 30 μM) of isoproterenol, and the cAMP responses measured. The increase in cAMP is expressed as a percentage, normalized to the cAMP response observed from β2-AR-mCer expressing cells at saturating concentration of isoproterenol. The cAMP response data were fit to a four-parameter logistic function to obtain dose-response curves and estimate potency (pEC_{50}). Values are mean ± S.D from at least 9 repeats across 3 experiments

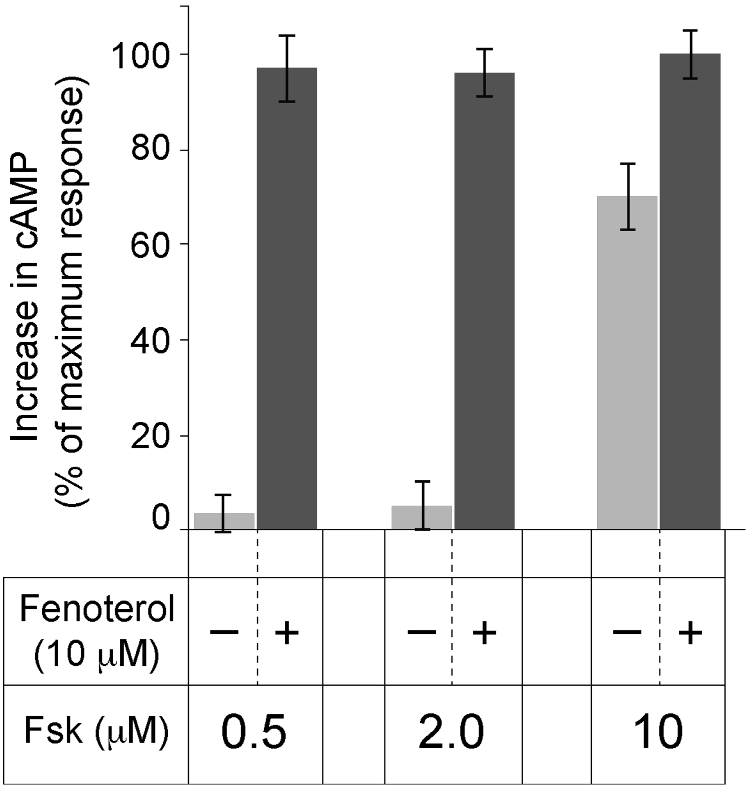

Supplementary Figure 5 - Forskolin synergizes signaling downstream of endogenous β*2-AR.* (a) cAMP accumulation in HEK293T cells over-expressing Gαq (mCer-tag), following stimulation with forskolin (Fsk) at either 2 μM or 10 μM, fenoterol (10 μM), or a combination of forskolin (2 μM) and fenoterol (10 μM).

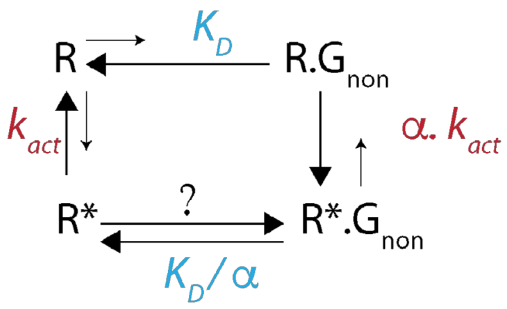

$$k_{act} = [R^*] / [R]$$

$$K_D = [R][G_{non}] / [R.G_{non}]$$

$$\alpha\, k_{act} = [R^*.G_{non}] / [R.G_{non}]$$

$$K_D/\alpha = [R^*][G_{non}] / [R^*.G_{non}]$$

Expressing in terms of R and G_{non}

$$[R^*] = k_{act} * [R]$$

$$[R.G_{non}] = [R][G_{non}] / K_D$$

$$[R^*.G_{non}] = \alpha.\, k_{act} [R][G_{non}] / K_D$$

If $R << G_{non}$, $G_{non} \sim G_t$ (total non cognate G protein)

$$[R^*] = k_{act} * [R]$$

$$[R.G_{non}] = [R][G_t] / K_D$$

$$[R^*.G_{non}] = \alpha.\, k_{act} [R][G_t] / K_D$$

Total receptor

$$R_t = R + R^* + R.G_{non} + R^*.G_{non}$$

$$[R_t] = [R] + (k_{act} * [R]) + ([R][G_t] / K_D) + (\alpha.\, k_{act} [R][G_t] / K_D)$$

$$[R_t] = [R] \{ 1 + k_{act} + ([G_t] / K_D) + (\alpha.\, k_{act} . [G_t] / K_D)\}$$

parameter estimation for fitting:

k_{act} = must be less than 1

G_t = 10 nM to 30 μM

K_D is estimated from Qp binding to β2-mCer

K_D = 100 μM

α = arbitary values between 0.01 and 100

Ratio of primed to total receptor is calculated as

$$R^*/R_t = (k_{act} * [R]) / [R] \{ 1 + k_{act} + ([G_t] / K_D) + (\alpha.\, k_{act} . [G_t] / K_D)\}$$

$$R^*/R_t = k_{act} / \{ 1 + k_{act} + ([G_t] / K_D) + (\alpha.\, k_{act} . [G_t] / K_D)\}$$

Supplementary Figure 6 – Equilibrium model of GPCR priming through non-cognate G protein interactions.

(a) Receptor-non-cognate G protein interaction modeled on the Ternary Complex Model of cognate G protein signalling. Receptor interactions with the G protein enhance transition to the primed state as modeled by the cooperativity factor (α). Fraction of receptor in the primed state (R^*/R_t) is calculated based on estimates of G protein concentration (10 nM to 30 μM) and binding affinity of the non-cognate G peptide (Qp) to β2-mCerulean membranes (100 μM).

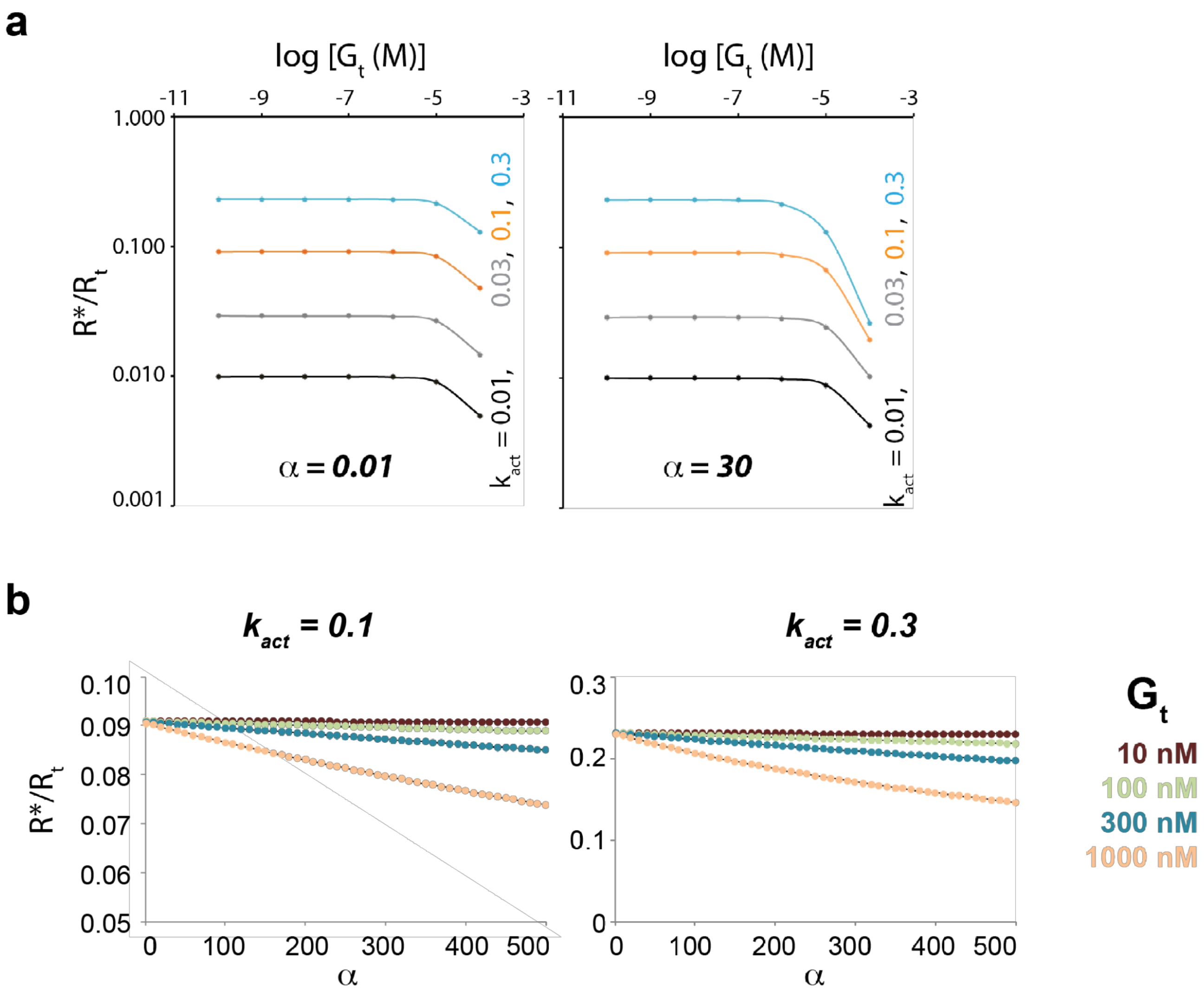

Supplementary Figure 7 – Equilibrium ternary complex model of GPCR signaling does not show augmented response in the presence of enhanced non-cognate G protein expression. Fraction of primed receptor (R^*/R_t) decreases with G protein expression, regardless of the fraction of spontaneously primed receptor (k_{act}; **a**) or cooperativity factor (α; **b**).

Supplementary Table 1

Three aspects of GPCR signaling that exhibit temporal persistence

(1) GPCR conformation – β_2-AR ICL3 sensor (β_2-AR conformation in parameter column);

(2) GPCR activation state – β_2-AR-Sp SPASM sensor (Tethered Sp interaction with β_2-AR); and

(3) G protein activation in *in vitro* reconstituted membranes (G protein activation). The details for each are tabulated

	**Parameter**	**Priming Peptide**	**Sensor used**	**Persistence time**		
				Minimum	Maximum	t½
1	Tethered Sp interaction with β_2-AR	bioQP	β_2-AR-Sp SPASM	90 s (1 ½ min)	150 s (2 ½ min)	330 s (5.6 min)
2	Tethered Sp interaction with β_2-AR	PC-Qp	β_2-AR-Sp SPASM	165 s (2¾ min)	240 s (4 min)	
4	β_2-AR conformation	bioQp	β_2-AR-ICL3	150 s (2½ min)	240 s (4 min)	
5	β_2-AR conformation	PC-Qp	β_2-AR-ICL3	105 s (1¾ min)	240 s (4 min)	97 s (1½ min)
6	G protein activation	PC-Qp	β_2-AR-cherry	60 s		

Supplementary Table 2

Sequences of Gα subunit c-terminus peptides with hot-spot substitutions highlighted in red.

Sp, DTENIRRVFNDCRDIIQRMHLRQYELL;.
Sp → Qp DTENIRRVFNDCRDIIQRMHLRQYNLL (E392N)
Qp, DTENIRFVFAAVKDTILQLNLKEYNLV
Qp → Sp DTENIRFVFAAVKDTIQQLNLKQYNLV (L349Q; E355Q)

Supplementary Table 2
Primers for creating the β_2-AR-ICL3 sensor

Inserting AgeI site in-frame with β_2-AR

AgeI insert FP	CCAGGTGGAGCAGGAT**ACCGGT**GGGCGGACGGGG
AgeI insert RP	CCCCGTCCGCCCACCGGTATCCTGCTCCACCTGG

PCR and insert mCer in-frame with β_2-AR into AgeI introduced above:

AgeImCer Flex FP	GATACCGGTtcctcaGGTtcctcagtgagcaagggcgaggagctg
AgeImCer Flex RP	CCGCCCaccggttgaggaACCtgaggaCTtgtacagctcgtccatg
