# Supplementary material for: Minute-scale persistence of a GPCR conformation state triggered by non-cognate G protein interactions primes signaling
Source: Nat Commun. 2019 Oct 23;10:4836. doi: 10.1038/s41467-019-12755-9 (PMC6811539; doi:10.1038/s41467-019-12755-9)
Supplement: Supplementary file 2 — Reporting Summary [file 41467_2019_12755_MOESM2_ESM.pdf]

natureresearch

Corresponding author(s): Dr. Sivaraj Sivaramakrishnan

Last updated by author(s): Sep 12, 2019

Reporting Summary

Nature Research wishes to improve the reproducibility of the work that we publish. This form provides structure for consistency and transparency in reporting. For further information on Nature Research policies, see Authors & Referees and the Editorial Policy Checklist .

Statistics

For all statistical analyses, confirm that the following items are present in the figure legend, table legend, main text, or Methods section.

n/a	Confirmed	
☐	☒	The exact sample size (*n*) for each experimental group/condition, given as a discrete number and unit of measurement
☐	☒	A statement on whether measurements were taken from distinct samples or whether the same sample was measured repeatedly
☐	☒	The statistical test(s) used AND whether they are one- or two-sided<br>*Only common tests should be described solely by name; describe more complex techniques in the Methods section.*
☒	☐	A description of all covariates tested
☒	☐	A description of any assumptions or corrections, such as tests of normality and adjustment for multiple comparisons
☐	☒	A full description of the statistical parameters including central tendency (e.g. means) or other basic estimates (e.g. regression coefficient) AND variation (e.g. standard deviation) or associated estimates of uncertainty (e.g. confidence intervals)
☐	☒	For null hypothesis testing, the test statistic (e.g. *F*, *t*, *r*) with confidence intervals, effect sizes, degrees of freedom and *P* value noted<br>*Give P values as exact values whenever suitable.*
☒	☐	For Bayesian analysis, information on the choice of priors and Markov chain Monte Carlo settings
☒	☐	For hierarchical and complex designs, identification of the appropriate level for tests and full reporting of outcomes
☒	☐	Estimates of effect sizes (e.g. Cohen's *d*, Pearson's *r*), indicating how they were calculated

Our web collection on statistics for biologists contains articles on many of the points above.

Software and code

Policy information about availability of computer code

Data collection	Commercial - FluorEssence V3.9 (Origin-based) from Horiba and LiCor Odyssey - ImageStudio.<br>Open-source - FIJI/ImageJ, ProteoWizard and mMass v5.5
Data analysis	Numerical values were exported and analyzed in Microsoft-Excel and Origin 9.0

For manuscripts utilizing custom algorithms or software that are central to the research but not yet described in published literature, software must be made available to editors/reviewers. We strongly encourage code deposition in a community repository (e.g. GitHub). See the Nature Research guidelines for submitting code & software for further information.

Data

Policy information about availability of data

All manuscripts must include a data availability statement. This statement should provide the following information, where applicable:

- Accession codes, unique identifiers, or web links for publicly available datasets
- A list of figures that have associated raw data
- A description of any restrictions on data availability

All data generated or analyzed during this study are included in this published article.
The source data underlying Figs 1 b-i, 2 b-d, 3 c, d, e, g, 4 c-e, 5 b, d-f, 6 b, c, 7 a, b, d
and supplementary figures 1 a-d, 2 a, b, 3 b, c, 4 b, c and 5 are provided as a Source Data file.

Field-specific reporting

Please select the one below that is the best fit for your research. If you are not sure, read the appropriate sections before making your selection.

Life sciences study design

All studies must disclose on these points even when the disclosure is negative.

Sample size	Experiment in a single 'n' was performed with either one passage of cells or membranes derived from the same batch of cells. All experiments were repeated at least three times with cells or membranes derived from three different cell passages. Within each experiment, samples were replicated as follows FRET - 5 times (5 agonist, 5 buffer), cAMP 4 times (4 agonist, 4 buffer).
Data exclusions	Integrity of membrane preparations was assayed from the response to isoproterenol (deltaFRET). Membranes that did not respond to agonist application (no deltaFRET response) were not used for further experiments. Other than that - All data generated or analyzed during this study are included in this published article.
Replication	The increased FRET response from Q-peptide has been replicated independently by a rotation student who has not joined the lab, and has no stake in the project.
Randomization	Allocation of membranes into 'agonist-treated' or 'buffer-treated' groups was random, applying a zig-zag geometery to tube arrangement.
Blinding	Cell lysates for Western blot were prepared and coded by one author, while the Western blot experiment was performed by a second author who was only told that samples were either 'a' or 'b'. Other experiments were not blinded.

Reporting for specific materials, systems and methods

We require information from authors about some types of materials, experimental systems and methods used in many studies. Here, indicate whether each material, system or method listed is relevant to your study. If you are not sure if a list item applies to your research, read the appropriate section before selecting a response.

Materials & experimental systems

n/a	Involved in the study	
☐	☒	Antibodies
☐	☒	Eukaryotic cell lines
☒	☐	Palaeontology
☒	☐	Animals and other organisms
☒	☐	Human research participants
☒	☐	Clinical data

Methods

n/a	Involved in the study	
☒	☐	ChIP-seq
☒	☐	Flow cytometry
☒	☐	MRI-based neuroimaging

Antibodies

Antibodies used	anti-Vinculin, Streptavidin conjugated to HRP
Validation	Vinculin Antibody in Western Blot (WB)<br>Western blot analysis of Vinculin in HeLa cell lysate using a Vinculin recombinant rabbit monoclonal antibody (Product # 700062) at a dilution of 0.5 µg/mL.<br>https://www.thermofisher.com/antibody/product/Vinculin-Antibody-clone-42H89L44-Monoclonal/700062<br>(figure 14 of 14 on website linked above)<br><br>Pierce™ High Sensitivity Streptavidin-HRP #21130<br>Receptor-expressing membranes (without added biotinylated Qpep) ie -control were probed with the same reagent as membrane samples treated with a known concentration of the biotinylated Qpep (Supplementary figure 3b). The control samples had a much lower signal than the peptide-treated samples.

Eukaryotic cell lines

Policy information about cell lines

Cell line source(s)	HEK293 T flpIn : from Invitrogen- thermofisher-Lifetech
Authentication	n/a (Source cells were used for 15 passages)
Mycoplasma contamination	n/a
Commonly misidentified lines<br>(See ICLAC register)	n/a
